# Supplementary material for: Lifetime cigarette smoking and chronic widespread and regional pain in later adulthood: evidence from the 1946 British birth cohort study
Source: BMJ Open. 2018 Aug 29;8(8):e021896. doi: 10.1136/bmjopen-2018-021896 (PMC6119432; doi:10.1136/bmjopen-2018-021896)
Supplement: Supplementary file 1 [file bmjopen-2018-021896supp001.pdf]

Supplementary information

Supplementary Table 1: Summary of smoking history data (i.e. those with at least 1 wave of smoking data and pain at age 68, n=2409)

	Men, N (%)	Women, N (%)	P value
**Ever smoker, N=2409**			
No	310 (26.8)	431 (34.5)	<.001
Yes	848 (73.2)	820 (65.6)	
**Smoking history at**			
**Age 20, n=2074**			
Current smoker	551 (55.8)	508 (46.8)	<.001
Ex-smoker	129 (13.1)	145 (13.4)	
Never	308 (31.2)	433 (39.9)	
**Age 25, N=1956**			
Current smoker	390 (42.4)	344 (33.2)	<.001
Ex-smoker	255 (27.8)	302 (29.1)	
Never	274 (29.8)	391 (37.7)	
**Age 31, N=2032**			
Current smoker	375 (38.5)	373 (35.3)	.001
Ex-smoker	336 (34.5)	318 (30.1)	
Never	263 (27)	367 (34.7)	
**Age 36, N=2129**			
Current smoker	301 (29.7)	298 (26.8)	.001

	Men, N (%)	Women, N (%)	P value
Ex-smoker	431 (42.5)	423 (38)	
Never	283 (27.9)	393 (35.3)	
**Age 43, N=2196**			
Current smoker	278 (26.6)	270 (23.5)	<.001
Ex-smoker	397 (38)	312 (27.1)	
Never	371 (35.5)	568 (49.4)	
**Age 53, N=2157**			
Current smoker	201 (19.9)	210 (18.3)	<.001
Ex-smoker	437 (43.2)	348 (30.4)	
Never	374 (37.0)	587 (51.3)	
**Age 60-64, N=2027**			
Current smoker	109 (11.4)	120 (11.2)	<.001
Ex-smoker	452 (47.3)	367 (34.3)	
Never	395 (41.3)	584 (54.5)	
**Age 68, N=2366**			
Current smoker	113 (9.9)	96 (7.8)	<.001
Ex-smoker	537 (47.1)	413 (33.7)	
Never	489 (48.9)	718 (58.5)	

Supplementary Table 2: Associations between lifetime smoking history (vs never smoker) and pain outcomes, n=1759 (complete case analysis)

	**CWP vs no pain**		**CRP vs no pain**		**Other pain vs no pain**	
Model	RRR (95% CI)	P value	RRR (95% CI)	P value	RRR (95% CI)	P value
Sex-adjusted						
Never smoker	1	.0028	1	.17	1	.18
Predominantly non-smoker	1.83 (1.22, 2.75)		1.14 (0.89 , 1.48)		1.11 (0.79, 1.57)	
Predominantly smoker	2.39 (1.49 , 3.84)		1.34 (0.97 , 1.84)		1.53 (1.02 , 2.29)	
Lifelong smoker	1.60 (0.75 , 3.46)		0.78 (0.45 , 1.37)		0.91 (0.45, 1.85)	
*Fully-adjusted**						
Never smoker	1	.024	1	.35	1	.23
Predominantly non-smoker	1.76 (1.15 , 2.70)		1.10 (0.84, 1.44)		1.09 (0.77, 1.55)	
Predominantly smoker	2.09 (1.26 , 3.47)		1.20 (0.86, 1.69)		1.52 (0.99, 2.31)	
Lifelong smoker	1.47 (0.65 , 3.31)		0.73 (0.41 , 1.31)		0.99 (0.48 , 2.06)	

*Adjusted for all covariates (sex, own occupational class, educational level, BMI, leisure time physical activity, alcohol intake, longstanding illness and symptoms of anxiety and depression.)

Supplementary Table 3: Hazard ratio for all-cause mortality by lifetime smoking history

	Sex adjusted		Adjusted*	
	HR (95% CI)	P value	HR (95% CI)	P value
Never smoker	1		1	
Predominantly non-smoker	1.21 (0.89 1.64)	.22	1.22 (0.90 – 1.65)	.21
Predominantly smoker	1.59 (1.14 – 2.22)	.007	1.52 (1.09 – 2.13)	.014
Lifelong smoker	5.12 (3.79 – 6.90)	<.001	4.71 (3.48 – 6.39)	<.001

*adjusted for sex, father's occupational class and own occupational class
