# Supplementary material for: Lifetime cigarette smoking and chronic widespread and regional pain in later adulthood: evidence from the 1946 British birth cohort study
Source: BMJ Open. 2018 Aug 29;8(8):e021896. doi: 10.1136/bmjopen-2018-021896 (PMC6119432; doi:10.1136/bmjopen-2018-021896)
Supplement: Author's manuscript [file bmjopen-2018-021896.draft_revisions.pdf]

BMJ Open is committed to open peer review. As part of this commitment we make the peer review history of every article we publish publicly available.

When an article is published we post the peer reviewers' comments and the authors' responses online. We also post the versions of the paper that were used during peer review. These are the versions that the peer review comments apply to.

The versions of the paper that follow are the versions that were submitted during the peer review process. They are not the versions of record or the final published versions. They should not be cited or distributed as the published version of this manuscript.

BMJ Open is an open access journal and the full, final, typeset and author-corrected version of record of the manuscript is available on our site with no access controls, subscription charges or pay-per-view fees (http://bmjopen.bmj.com).

If you have any questions on BMJ Open's open peer review process please email info.bmjopen@bmj.com

BMJ Open

# Lifetime cigarette smoking and chronic widespread and regional pain in later adulthood: Evidence from the 1946 British birth cohort study

| | |
|---|---|
| Journal: | *BMJ Open* |
| Manuscript ID | bmjopen-2018-021896 |
| Article Type: | Research |
| Date Submitted by the Author: | 23-Feb-2018 |
| Complete List of Authors: | Bendayan, Rebecca; University College London, Population, Policy and Practice Programme<br>Cooper, Rachel; UCL, MRC Unit for Lifelong Health and Ageing at UCL<br>Muthuri, Stella; MRC Unit for Lifelong Health and Ageing, |
| Keywords: | Chronic Pain, Cigarette Smoking, Life Course Epidemiology, Birth Cohort |
| | |

SCHOLARONE™ Manuscripts

For peer review only

**Lifetime cigarette smoking and chronic widespread and regional pain in later adulthood: Evidence from the 1946 British birth cohort study**

Rebecca Bendayan[1;2], Rachel Cooper[1], Stella G Muthuri[1]

[1] MRC Unit for Lifelong Health and Ageing at UCL, 33 Bedford Place, London, WC1B 5JU, UK

[2]Population, Policy and Practice Programme, UCL Institute of Child Health, 30 Guilford Street, London, WC1N 1EH UK, United Kingdom.

Correspondence to: Rebecca Bendayan, r.bendayan@ucl.ac.uk

The Corresponding Author has the right to grant on behalf of all authors and does grant on behalf of all authors, a worldwide licence to the Publishers and its licensees in perpetuity, in all forms, formats and media (whether known now or created in the future), to i) publish, reproduce, distribute, display and store the Contribution, ii) translate the Contribution into other languages, create adaptations, reprints, include within collections and create summaries, extracts and/or, abstracts of the Contribution, iii) create any other derivative work(s) based on the Contribution, iv) to exploit all subsidiary rights in the Contribution, v) the inclusion of electronic links from the Contribution to third party material where-ever it may be located; and, vi) licence any third party to do any or all of the above.

Key words: Chronic Pain; Cigarette Smoking; Life Course Epidemiology; Birth Cohort



For peer review only - http://bmjopen.bmj.com/site/about/guidelines.xhtml

**Abstract**

**Objective:** To examine whether different lifetime patterns of cigarette smoking are associated with chronic widespread and regional pain at age 68.

**Design:** Prospective cohort study

**Setting:** England, Scotland and Wales

**Participants:** Up to 2347 men and women from the Medical Research Council National Survey of Health and Development, who have been followed up since birth in 1946, have information on cigarette smoking status across adulthood and whose experience of pain was assessed at age 68.

**Primary outcome measures:** Pain was self-reported at age 68, and chronic widespread pain (CWP) was defined according to American College of Rheumatology criteria. Participants who reported having pain for at least 3 months but who did not meet the CWP definition were classified as having chronic regional pain (CRP); those who reported pain which had lasted for less than 3 months were classified as having 'other' pain; and the reference group was those who did not report pain.

**Results:** Findings from multinomial logistic regression models indicated that compared with never smokers, predominantly non-smokers, predominantly smokers and lifelong smokers all had an increased risk of CWP; Relative-Risk-Ratios = 1.70 [95%CI 1.16 – 2.49]; 2.10 [95%CI 1.34 – 3.28] and 1.88 [95%CI 0.99 – 3.57], respectively, after adjusting for sex, own occupational class, educational level, body mass index, leisure time physical activity, alcohol intake, longstanding illness and symptoms of anxiety and depression. No association was observed between lifetime smoking history and CRP or other pain.

**Conclusions:** These results suggest that exposure to cigarette smoking at any stage in adulthood was associated with higher risk of CWP in later adulthood; highlighting the ongoing importance of smoking prevention programmes. It also suggests that assessment of



For peer review only - http://bmjopen.bmj.com/site/about/guidelines.xhtml

lifetime smoking behaviour may be more useful in identifying those at greater risk of CWP in later life than assessment of current smoking status.

**Strengths and limitations of this study**

This study uses prospective data on smoking behaviour ascertained across adulthood (from age 20 to 68) providing us with the opportunity to characterise different lifetime patterns of smoking.

- This study distinguishes chronic widespread pain from regional pain and examines the risk of both.
- Chronic pain was assessed at a single time point so we were not able to establish temporality.
- Lifelong smokers were under-represented in the study sample as they had greater rates of all-cause mortality.
- Despite losses to follow up over the 69 years of the study, the sample has remained broadly representative of the national population of England, Scotland and Wales born at a similar time.

For peer review only



For peer review only - http://bmjopen.bmj.com/site/about/guidelines.xhtml

## Introduction

Chronic pain is highly prevalent in older populations and has major adverse impacts on quality of life, wellbeing and activity participation[1-4]. Identifying modifiable risk factors associated with pain may inform interventions to prevent and/or alleviate its consequences in later life. Cigarette smoking is one of many modifiable behavioural risk factors that may play a role in the development and progression of chronic pain. While this risk factor has already been widely studied[5,6] with most longitudinal studies reporting weak or null associations[7-10], the majority of these have been in populations younger than 65.

Moreover, even when studies have included older adults these have usually only considered a single assessment of smoking status (either at baseline or at the current or most recent time point). This information has then been used to classify individuals as current, former or never smokers, or as ever or never smokers. This is unlikely to adequately capture the variability in lifetime exposure to smoking and its associated health risks due to changes in smoking behaviour across adulthood. Distinguishing people with different lifetime smoking histories and investigating differences in their health prospects may be especially important in older adults; although the prevalence of smoking has steadily declined across all age groups over the last few decades, the baby boom generation who are currently reaching old age were at high risk of exposure to smoking earlier in adulthood[11]. In addition, they are likely to have been exposed to anti-smoking campaigns and comprehensive tobacco control policies early enough in adulthood to have potentially benefited from the subsequent reductions in risk of smoking related diseases linked to premature mortality[12]. While people with some history of smoking are therefore now more likely to reach old age than they would have been in previous generations, whether they reach old age with more chronic conditions and symptoms such as pain remains to be established.



For peer review only - http://bmjopen.bmj.com/site/about/guidelines.xhtml

Using data from the MRC National Survey of Health and Development (NSHD), which has assessed smoking status at multiple time points across adulthood, this paper aims to examine whether different lifetime patterns of cigarette smoking are associated with chronic widespread and regional pain at age 68.

## Methods

### Study population

The MRC NSHD is a socially stratified population sample of 5362 single, legitimate births that occurred in England, Wales and Scotland in one week of March 1946. Participants have been prospectively followed from birth, and participation rates have remained high[13-16]. At the 24th data collection, conducted between 2014 and 2015 when study members were aged 68-69, study participants were first asked to complete a postal questionnaire at age 68 before then being invited to have a home visit by a research nurse at age 69[15]. Of the 2816 people in the target sample living in England, Scotland and Wales, 2370 (84.2%) completed a postal questionnaire. In addition, a postal questionnaire was sent to 126 study members living abroad who remain in contact with the study of whom 83 (65.9%) returned a questionnaire. No attempt was made to contact the remaining 2420 study members: 957 (17.8%) had already died, 620 (11.6%) had previously withdrawn from the study, 448 (8.3%) had emigrated and were no longer in contact with the study, and 395 (7.4%) had been untraceable for more than five years.

Ethical approval for the data collection at age 68-69 was obtained from the Queen Square Research Ethics Committee (14/LO/1073) and the Scotland A Research Ethics Committee (14/SS/1009) and written informed consent was obtained from study members.



For peer review only - http://bmjopen.bmj.com/site/about/guidelines.xhtml

**Assessment of pain**

Information on pain was ascertained in the postal questionnaire administered at age 68. Participants were asked whether they had experienced any ache or pain in the last month which had lasted for one day or longer, not including pain occurring during the course of a feverish illness such as flu. Those who reported pain were then asked to report whether or not they had experienced this pain for at least 3 months and to shade the location of their pain using a four-view body manikin. Using American College of Rheumatology criteria[17] chronic widespread pain (CWP) was defined as pain present for 3 months or longer, both above and below the waist; on both the left and right side of the body; and in the axial skeleton. Participants who reported having pain for at least 3 months but who did not meet the CWP definition were classified as having chronic regional pain (CRP) and those who reported pain that had lasted for less than 3 months were classified as having 'other' pain[18].

**Assessment of smoking**

Data on cigarette smoking were obtained on eight occasions across adulthood using postal questionnaires (at ages 20, 25, 31, 60-64, 68-69) and nurse interviews (at ages 36, 43, 53). At each of these ages, study members were asked whether they currently smoked cigarettes. Participants who provided a negative response were asked whether they had ever smoked. This information was used to construct a set of variables indicating smoking status at each age from 20 to 68, coded as current, former and never smoker. Using these 3-category variables, a lifetime smoking history variable was then constructed according to Clennell et al.[19]. Using this approach, we included those who provided smoking data for at least 3 waves and for whom missing data were not sequential at 3 or more waves (n=3705). Based on their reported smoking status at each age across adulthood, participants were grouped into one of



For peer review only - http://bmjopen.bmj.com/site/about/guidelines.xhtml

four categories to summarise their smoking history: (1) participants who reported being non-smokers at all available data collections were classified as 'never smokers'; (2) participants who reported being smokers at all available data collections were classified as 'lifelong smokers'; participants who were classified as smokers at some ages and as ex or never smokers at other ages were classified as: (3) 'predominantly non-smoker' if they were a non-smoker on at least 3 occasions and more often reported being a non-smoker than a smoker; (4) 'predominantly smoker' if they reported being a smoker on 4 or more occasions and more often reported being a smoker than a non-smoker. For example, if a study member reported being a smoker on five occasions and an ex-smoker on two occasions, they were classified as 'predominantly smoker' whereas if they reported the reverse they were classified as 'predominantly non-smoker'[19].

**Covariates**

Potential confounders and mediators were selected a priori based on previous research on chronic pain and health behaviours[20-23]. These were sex, socioeconomic position (indicated by own occupational class and educational level), body mass index (BMI), other health behaviours (leisure time physical activity and alcohol intake), longstanding illness and symptoms of anxiety and depression. Unless otherwise specified these were assessed at age 68. Own occupation at age 53 years (or if not available, the most recent measure in adulthood) was categorised according to the Registrar General's social classification into three groups: high (I or II); middle (III non-manual [NM] or III manual [M]); low (IV or V). Highest educational level achieved by age 26 was categorised into three groups: no qualifications; up to O-level or equivalent; A level or equivalent and above. BMI ($kg/m^2$) was calculated from height (m) and weight (kg) measured by a trained nurse during the clinical assessment at age 69. Physical activity was assessed through self-reports of the level



For peer review only - http://bmjopen.bmj.com/site/about/guidelines.xhtml

For peer review only

of participation in sports, vigorous leisure activities or exercise grouped as: inactive, moderately active (1-4 times/month) or regularly active (5 times or more/month). Similarly, alcohol intake in the last year was self-reported as never, only on special occasions, monthly or less, 2-4 times per month, 2-3 times per week, or 4 or more times per week. In addition, participants were asked whether they had any longstanding illness or health problems which had lasted, or were expected to last for 6 months or more. Symptoms of anxiety and depression were assessed using the 28-item General Health Questionnaire (GHQ-28). Each GHQ-28 item was coded using the General Scoring Method (i.e. 0 for response choices 1 and 2; and as 1 or response choices 3 and 4). Symptoms of anxiety and depression were indicated by summing responses to all items and applying a threshold for caseness of 5 or more[24].

**Statistical analyses**

Multinomial logistic regression models were used to examine the associations between lifetime smoking history and pain outcomes (with no pain as the reference category). Models were first adjusted for sex and formal tests of interaction between sex and lifetime smoking history were undertaken. As no evidence of interactions with sex were found, subsequent models were sex adjusted. We then adjusted for each set of covariates in turn before adjusting for all covariates simultaneously in a final model. All models included the maximum number of participants with complete data on lifetime smoking history and pain (n=2347). To reduce data loss, covariates with missing values (i.e. educational attainment [n=114], occupational class [n=6], BMI [n=435], leisure time physical activity [n=25], alcohol intake [n=25], longstanding illness or health problems [n=24] and GHQ-28 [n=422] were imputed using multiple imputation by chained equations[25]. Analyses were performed across 20 imputed datasets and combined using Rubin's rules. In sensitivity analyses, models were rerun on the



For peer review only - http://bmjopen.bmj.com/site/about/guidelines.xhtml

sample with complete data on all covariates (n=1759). All analyses were conducted using STATA v14.1.

## Results

Of the total analytical sample of 2347, 1304 (55.6%) reported pain, with 10.6% reporting CWP, 30.3% reporting CRP, and 14.7% reporting 'other pain'. More women than men reported CWP (13.2% for women vs 7.7% for men, p<.001). Overall, the prevalence of smoking declined across adulthood from 55.8% for men and 46.8% of women at age 20 to 9.9% for men and 7.8% for women by age 68 (p-values for sex differences at both ages<.001) (Supplementary Table 1). The prevalence of lifelong non-smokers was 34.3% for women and 26.3% for men. Of those classified as predominantly non-smokers, 61.6% had stopped smoking at least once by age 25 and only 0.5% were smokers at the most recent report. Of those classified as predominantly smokers, 88.9% were smokers at age 25, but only 14.2% were still smokers at the most recent report. Descriptive statistics are shown in Table 1.

PLEASE, INSERT TABLE 1 AROUND HERE

In sex-adjusted models, predominantly non-smokers, predominantly smokers, and lifelong smokers all had a higher likelihood of CWP, compared with lifelong non-smokers (Relative-Risk Ratios (RRR) of CWP vs no pain=1.80 (95%CI 1.26 – 2.58), 2.64 (95%CI 1.74 – 3.99) and 2.27 (95%CI 1.25 – 4.13), respectively (Table 2, Model 1). Adjustment for each set of covariates partially attenuated associations (especially for those classified as predominantly smoker and lifelong smoker), with longstanding illness having the greatest impact on effect estimates. However, associations were maintained in fully-adjusted models; RRRs = 1.70 (95%CI 1.16 – 2.49) for predominantly non-smokers, 2.10 (95%CI 1.34 – 3.28) for predominantly smokers and 1.88 (95%CI 0.99 – 3.57) for lifelong smokers (Table 2, Model



For peer review only - http://bmjopen.bmj.com/site/about/guidelines.xhtml

6). No associations were observed between lifetime smoking history and CRP or other pain (Table 2).

PLEASE, INSERT TABLE 1 AROUND HERE

When analyses were restricted to those with complete data (n=1759), we found similar results and the overall conclusions remained the same (Supplemental Table 2).

## Discussion

The main aim of this study was to examine whether different patterns of cigarette smoking over the life course were associated with CWP and CRP at age 68 in a nationally representative birth cohort. Our results showed that exposure to cigarette smoking at any time point across adulthood was associated with increased risk of CWP at age 68, even after adjustment for a range of potential confounders and mediators. However, there was no evidence of associations between lifetime cigarette smoking and CRP or other pain.

*Comparisons with other studies*

Our finding of an association between different patterns of lifetime smoking and CWP in older adults is in contrast with some other previous studies of chronic pain in older adults. For example, in a longitudinal study of British adults aged 50 years or older, there was no evidence that former or current smokers had an increased likelihood of new-onset widespread pain over 3 years of follow-up when compared with never smokers[23]. Likewise, a cross-sectional study found that smoking status (classified as non-smoker, former smoker, occasional smoker or daily smoker) was not associated with chronic pain among people aged 65 years and older in Sweden[26]. Our findings may differ because of the way in which smoking behaviour was characterised over time. By using data collected prospectively across adulthood we were able to distinguish different lifetime patterns of cigarette smoking. This



For peer review only - http://bmjopen.bmj.com/site/about/guidelines.xhtml

has strengths when compared with other studies that have relied on single assessments of smoking and may therefore not adequately or reliably capture all relevant lifetime exposure.

*Potential explanations of findings*

Our finding of increased risk of CWP among all smoking groups (i.e. predominantly non-smokers, predominantly smokers and lifelong smokers), when compared with never smokers suggests that cigarette smoking even for a short period of time over the life course may have a long-lasting impact on the probability of reporting CWP in later adulthood. One plausible explanation for this finding is that smoking may produce changes in central nervous system function that increase susceptibility to pain the effects of which continue to persist after smoking cessation[27]. Another consideration isthat the associations between smoking and pain may be bi-directional; not only is there evidence that tobacco smoking may be a risk factor for the development and exacerbation of pain but also that pain may serve to motivate smoking[7]. For this reason, it is possible that some individuals in our study may have developed CWP in early adulthood and used smoking as a coping strategy for this with this then causing them to experience greater difficulty quitting[28].

That associations were observed between lifetime smoking and CWP but not with CRP or other pain suggests that any influence of smoking on pain is likely to be operating via mechanisms related specifically to the development of CWP. This is consistent with the idea that CWP and CRP may have different underlying aetiologies[7]. For example, some studies have suggested that nicotine may alter pain regulatory mechanisms (e.g. diminished pain sensitivity or cause structural changes to other systems) which might have long-term consequences for painful chronic conditions[29]. The associations between lifetime smoking and CWP were only partially attenuated after adjustment for a range of factors including sex, socioeconomic position, body size, other health behaviour, longstanding illness and



For peer review only - http://bmjopen.bmj.com/site/about/guidelines.xhtml

symptoms of anxiety and depression. This suggests that there might be other relevant explanatory variables. For example, although affective symptoms have been taken into consideration in our study some psychosocial models have highlighted the role of pain-related stress, fear or anxiety sensitivity in the transition from acute to chronic pain[7,30,31]. These pain specific measures were not available in our data and therefore future studies are encouraged to include these and examine their association with CWP onset and smoking.

*Methodological considerations*

One of the main strengths of our study was the use of data from a large nationally representative sample of older adults with prospective ascertainment of smoking status across adulthood which allowed us to characterise different lifetime patterns of smoking. While the use of recalled information on quitting attempts between assessments could have introduced bias, when we performed sensitivity analyses to check for potential inconsistencies in reporting and compared results with retrospective measures on time since quitting for former smokers there were no differences in findings. However, these analyses could be affected by survival bias as lifelong smokers were under-represented in the study sample as they had greater rates of all-cause mortality across adulthood up to age 69.

Another potential limitation of our analyses is that we cannot rule out reverse causation as chronic pain was only assessed at one time point. As noted above, recent research has highlighted that the association between smoking and pain is likely to be bi-directional[28,32] and so future longitudinal studies with prospective ascertainment of both smoking and pain across life should be performed. Although our findings are generalizable to the current generation of adults reaching old age in the UK, changing trends in smoking behaviour over the last 70 years[11,33], should be considered and further research is needed to examine whether similar associations will be observed in more recently born cohorts who will have



For peer review only - http://bmjopen.bmj.com/site/about/guidelines.xhtml

experienced different selection effects into smoking and different lifetime patterns of exposure to smoking.

*Conclusions*

Our results highlight the role of smoking at any time point across adulthood as an independent risk factor for CWP in later adulthood, after considering socio-economic, psychosocial and health-related factors. The evidence provided for the long-lasting effect of smoking on this condition highlights the potential benefit of considering smoking history rather than current smoking status when identifying those at greater risk of CWP in later life. These findings are relevant from a clinical and public health perspective, as smoking is a modifiable risk factor. Further research focusing on pain and smoking prevention and intervention initiatives should be performed.

For peer review only



For peer review only - http://bmjopen.bmj.com/site/about/guidelines.xhtml

**Acknowledgements:** The authors are grateful to NSHD study members for their continuing support. We also thank members of the NSHD scientific and data collection teams who have been involved in NSHD data collections.

**Funding:** This work was supported by the UK Medical Research Council who provide support for RC and SM (Programme code MC_UU_12019/4) and fund the MRC National Survey of Health and Development. RB was supported by a grant from the Spanish Ministry of Science and Innovation (PSI2013-42512- P). The funders of the study had no role in the study design, data collection, data analysis, data interpretation, writing of the report or the decision to submit the article for publication.

**Open access:** This is an Open Access article distributed in accordance with the terms of the Creative Commons Attribution (CC BY 3.0) license, which permits others to distribute, remix, adapt and build upon this work, for commercial use, provided the original work is properly cited. See: http://creativecommons.org/licenses/by/3.0.

**Declaration of competing interests:** All authors have completed the ICMJE uniform disclosure form at www.icmje.org/coi_disclosure.pdf and declare: RC and SM received financial support from the UK Medical Research Council for the submitted work; no financial relationships with any organisations that might have an interest in the submitted work in the previous three years; no other relationships or activities that could appear to have influenced the submitted work.

**Author contributions:** RB and SGM conceived the idea for this study and along with RC developed the study objectives. RC was part of the team responsible for the 68-69 year data collection. RB and SGM undertook statistical analyses. All authors contributed to the interpretation of the data. RB and SGM drafted the article and all authors contributed to its critical revision and provided final approval of the version to be published. RB and SGM are



For peer review only - http://bmjopen.bmj.com/site/about/guidelines.xhtml

the guarantors and accept full responsibility for the work and the conduct of the study, had access to the data, and controlled the decision to publish. All authors had full access to all of the data (including statistical reports and tables) in the study and can take responsibility for the integrity of the data and the accuracy of the data analysis.

**Transparency declaration:** RB and SGM affirm that the manuscript is an honest, accurate, and transparent account of the study being reported and that no important aspects of the study have been omitted.

**Data sharing statement:** Data are available on request to the NSHD Data Sharing Committee. NSHD data sharing policies and processes meet the requirements and expectations of MRC policy on sharing of data from population and patient cohorts: http://www.mrc.ac.uk/research/research-policy-ethics/data-sharing/policy/. Data requests should be submitted to mrclha.swiftinfo@ucl.ac.uk ; further details can be found at http://www.nshd.mrc.ac.uk/data.aspx DOI: 10.5522/NSHD/Q101;10.5522/NSHD/Q102; 10.5522/NSHD/Q103.

These policies and processes are in place to ensure that the use of data from this 71 year old national birth cohort study is within the bounds of consent given previously by study members, complies with MRC guidance on ethics and research governance, and meets rigorous MRC data security standards.

For peer review only

**References:**


1. Gardener EA, Huppert FA, Guralnik JM, Melzer D. Middle□Aged and Mobility□ Limited: Prevalence of Disability and Symptom Attributions in a National Survey. *J Gen Intern Med* 2006;*21*(10):1091-1096. doi:10.1111/j.1525-1497.2006.00564.x
2. Leveille SG, Bean J, Ngo L, *et al.* (2007). The pathway from musculoskeletal pain to mobility difficulty in older disabled women. *Pain* 2007;*128*(1): 69-77. doi:10.1016/j.pain.2006.08.031
3. Leveille SG, Fried L, Guralnik JM. Disabling symptoms. *J Gen Intern Med* 2002;*17*(10):766-773. doi:10.1046/j.1525-1497.2002.20229.x
4. Patel KV, Guralnik JM, Dansie EJ, Turk DC. Prevalence and impact of pain among older adults in the United States: findings from the 2011 National Health and Aging Trends Study. *Pain* 2013;*154*(12):2649-2657. doi:10.1016/j.pain.2013.07.029
5. McCarthy LH, Bigal ME, Katz M*, et al.* Chronic pain and obesity in elderly people: results from the Einstein aging study. *J Am Geriatr Soc* 2009;*57*(1):115-119. doi:10.1111/j.1532-5415.2008.02089.x
6. Mork PJ, Vasseljen O, Nilsen TI. Association between physical exercise, body mass index, and risk of fibromyalgia: Longitudinal data from the Norwegian Nord□Trøndelag Health Study. *Arthritis Care Res* 2010;*62*(5):611-617. doi:10.1002/acr.20118
7. Ditre JW, Brandon TH, Zale EL, Meagher MM. Pain, nicotine, and smoking: research findings and mechanistic considerations. *Psychol Bull* 2011;*137*(6):1065. doi:10.1037/a0025544
8. Parkerson HA, Zvolensky MJ, Asmundson GJ. Understanding the relationship between smoking and pain. *Expert Rev Neurother* 2013;*13*(12):1407-1414. doi:10.1586/14737175.2013.859524


For peer review only



For peer review only - http://bmjopen.bmj.com/site/about/guidelines.xhtml

9. Shiri R, Karppinen J, Leino-Arjas P, *et al.* The Association between Smoking and Low Back Pain: A Meta-analysis. *Am J Med* 2010;*123*(1):87.e7-87.e35. doi:10.1016/j.amjmed.2009.05.028

10. Mundal I, Gråwe R, Bjørngaard J, *et al.* Prevalence and long-term predictors of persistent chronic widespread pain in the general population in an 11-year prospective study: the HUNT study. *BMC Musculoskelet Disord* 2014, 15(1), 213. doi:10.1186/1471-2474-15-213

11. Davy M. Time and generational trends in smoking among men and women in Great Britain, 1972–2004/05. *Health Statistics Quarterly* 2006;*32*:35-43.

12. Pirie K, Peto R, Reeves GK, *et al.* The 21st century hazards of smoking and benefits of stopping: a prospective study of one million women in the UK. *Lancet* 2013;*381*(9861):133-141. doi:10.1016/S0140-6736(12)61720-6

13. Wadsworth M, Kuh D, Richards M, Hardy R. Cohort Profile: The 1946 National Birth Cohort (MRC National Survey of Health and Development). *Int J Epidemiol* 2006;35(1):49-54. doi:10.1093/ije/dyi201

14. Kuh D, Pierce M, Adams J, *et al.* Cohort profile: updating the cohort profile for the MRC National Survey of Health and Development: a new clinic-based data collection for ageing research. *Int J Epidemiol* 2011;40(1):e1-9. doi:10.1093/ije/dyq231

15. Kuh D, Wong A, Shah I, *et al.* The MRC National Survey of Health and Development reaches age 70: maintaining participation at older ages in a birth cohort study. *Eur J Epidemiol* 2016;31(11):1135-1147. doi:10.1007/s10654-016-0217-8

16. Stafford M, Black S, Shah I, *et al.* Using a birth cohort to study ageing: representativeness and response rates in the National Survey of Health and Development. *Eur J Ageing* 2013;*10*(2):145-157. doi:10.1007/s10433-013-0258-8



For peer review only - http://bmjopen.bmj.com/site/about/guidelines.xhtml

17. Wolfe F, Smythe HA, Yunus MB, *et al.* The American College of Rheumatology 1990 criteria for the classification of fibromyalgia. *Arthritis & Rheumatology* 1990;*33*(2): 160-172. doi:10.1002/art.1780330203

18. Muthuri SG, Kuh D, Bendayan R, *et al*. Chronic physical illness in early life and risk of chronic widespread and regional pain at age 68: evidence from the 1946 British birth cohort. *Pain* 2016;*157*(10):2382. doi:10.1097/j.pain.0000000000000663

19. Clennell S, Kuh D, Guralnik J, *et al*. Characterisation of smoking behaviour across the life course and its impact on decline in lung function and all-cause mortality: evidence from a British birth cohort. *J Epidemiol Community Health* 2008;*62*(12):1051-1056. doi:10.1136/jech.2007.068312

20. Bergman S. Psychosocial aspects of chronic widespread pain and fibromyalgia. *Disability and Rehabilitation* 2005;*27*(12):675-683. doi:10.1080/09638280400009030

21. Ferreira PH, Beckenkamp P, Maher CG, *et al.* (2013). Nature or nurture in low back pain? Results of a systematic review of studies based on twin samples. *Eur J Pain* 2013;*17*(7):957-971. doi:10.1002/j.1532-2149.2012.00277.x

22. Junqueira DRG, Ferreira ML, Refshauge K, *et al.* Heritability and lifestyle factors in chronic low back pain: results of the Australian Twin Low Back Pain Study (The AUTBACK study). *Eur J Pain* 2014;*18*(10):1410-1418. doi:10.1002/ejp.506

23. McBeth J, Lacey RJ, Wilkie R. Predictors of New□Onset Widespread Pain in Older Adults: Results From a Population□Based Prospective Cohort Study in the UK. *Arthritis & Rheumatology* 2014;*66*(3):757-767. doi:10.1002/art.38284

24. Goldberg DP, Hillier VF. A scaled version of the General Health Questionnaire. *Psychol Med* 1979;9(1):139-145. doi:10.1017/S0033291700021644

25. Sterne JAC, White IR, Carlin JB, *et al.* Multiple imputation for missing data in epidemiological and clinical research: potential and pitfalls. *BMJ* 2009;338.



For peer review only - http://bmjopen.bmj.com/site/about/guidelines.xhtml

26. Jakobsson U, Larsson C. Smoking and chronic pain among people aged 65 years and older. *Pain Practice* 2014;*14*(3):237-244. doi:10.1111/papr.12067

27. Perkins KA, Grobe JE, Stiller RL, *et al.* Effects of nicotine on thermal pain detection in humans. *Exp Clin Psychopharmacol*;2:95-106.

28. Ditre JW, Kosiba JD, Zale EL, *et al.* Chronic pain status, nicotine withdrawal, and expectancies for smoking cessation among lighter smokers. *Ann Behav Med* 2016;*50*(3):427-435. doi:10.1007/s12160-016-9769-9

29. Shi Y, Weingarten TN, Mantilla CB, *et al.* Smoking and Pain Pathophysiology and Clinical Implications. *The Journal of the American Society of Anesthesiologists* 2010;*113*(4): 977-992.

30. Asmundson GJ, Norton PJ, Vlaeyen JW. Fear-avoidance models of chronic pain: an overview. *Understanding and Treating Fear of Pain* 2004;3-24.

31. Vlaeyen JW, Linton SJ. Fear-avoidance and its consequences in chronic musculoskeletal pain: a state of the art. *Pain* 2000;*85*(3):317-332. doi:10.1016/S0304-3959(99)00242-0

32. Zale EL, Maisto SA, Ditre JW. Anxiety and depression in bidirectional relations between pain and smoking: Implications for smoking cessation. *Behavior Modification* 2016;*40*(1-2):7-28. doi:10.1177/0145445515610744

33. Thun MJ, Carter BD, Feskanich D, et al. 50-year trends in smoking-related mortality in the United States. *N Engl J Med* 2013;*368*(4):351-364. doi:10.1056/NEJMsa1211127

For peer review only



For peer review only - http://bmjopen.bmj.com/site/about/guidelines.xhtml

Table 1: Characteristics of participants from the MRC National Survey of Health and Development by sex at age 68 [Maximum N=2347]

| | Men, N† (%) | Women, N† (%) | P value* |
|---|---|---|---|
| Total | 1,123 (47.9) | 1,224 ( 52.1) | |
| **Pain at age 68** | | | |
| No pain | 537 (47.8) | 506 (41.3) | <.001 |
| Other pain | 184 (16.4) | 162 (13.2) | |
| Chronic Regional Pain | 316 (28.1) | 394 (32.2) | |
| Chronic Widespread Pain | 86 (7.7) | 162 (13.2) | |
| **Lifetime smoking history** | | | |
| Never smoker | 295 (26.3) | 420 (34.3) | <.001 |
| Predominantly non-smoker | 527 (46.9) | 516 (42.2) | |
| Predominantly smoker | 228 (20.3) | 224 (18.3) | |
| Lifelong smoker | 73 (6.5) | 64 (5.2) | |
| **BMI (kg/m²) at age 69** (n=1919) | | | |
| Mean (SD) | 28.0 (4.46) | 28.1 (5.56) | .92 |
| **Occupational class at age 53** | | | |
| High (I/II) | 631 (56.3) | 458 (37.5) | <.001 |
| Middle (IIINM/ IIIM) | 385 (34.4) | 543 (44.5) | |
| Low (IV/V) | 104 (9.3) | 220 (18.0) | |
| **Educational attainment by age 26** | | | |
| A level or equivalent & above | 505 (47.2) | 374 (32.2) | <.001 |
| Up to O level or equivalent | 212 (19.8) | 419 (36.1) | |
| None | 354 (33.1) | 369 (31.8) | |
| **Alcohol intake at age 68** | | | |
| Never/I drunk in the past | 205 (18.5) | 396 (32.7) | <.001 |
| Monthly or less | 55 (5.0) | 107 (8.8) | |
| 2-4 times per month | 157 (14.1) | 203 (16.8) | |
| 2-3 times per week | 295 (26.6) | 262 (21.6) | |
| ≥4 times per week | 398 (35.9) | 244 (20.1) | |
| **Participation in leisure time physical activity at age 68** | | | |
| More active( ≥5 times/ month) | 319 (28.7) | 320 (26.4) | .18 |
| Less active ( 1-4 times/ month) | 123 (11.1) | 161 (13.3) | |
| Inactive | 669 (60.2) | 730 (60.3) | |
| **Longstanding illness or health problem at age 68** | | | |



For peer review only - http://bmjopen.bmj.com/site/about/guidelines.xhtml

| | Men, N† (%) | Women, N† (%) | P value* |
|---|---|---|---|
| No | 449 (40.3) | 508(42.0) | .38 |
| Yes | 666 (59.7) | 700 (57.9) | |
| **Symptoms of anxiety and depression (GHQ-28) at age 68** | | | |
| No (≤4) | 840 (90.7) | 829 (83.0) | <.001 |
| Yes (>4) | 86 (9.3) | 170 (17.0) | |

*comparison of sexes using student t-test or chi-square tests as appropriate

† Sample restricted to those participants with data on smoking history and pain. N varies due to missing data on covariates

For peer review only

Table 2. Associations between lifetime smoking history and pain at age 68 (n=2347)

| | **CWP vs no pain** | | **CRP vs no pain** | | **Other pain vs no pain** | |
|---|---|---|---|---|---|---|
| | RRR (95% CI) | P | RRR (95% CI) | P | RRR (95% CI) | P |
| **Model 1** | | | | | | |
| Never smoker | 1 | .0001 | 1 | .25 | 1 | .25 |
| Predominantly non-smoker | 1.80 (1.26 , 2.58) | | 1.08 (0.86 , 1.35) | | 1.14 (0.85 , 1.52) | |
| Predominantly smoker | 2.64 (1.74 , 3.99) | | 1.32 (0.99 , 1.74) | | 1.44 (1.01 , 2.04) | |
| Lifelong smoker | 2.27 (1.25 , 4.13) | | 0.97 (0.62 , 1.51) | | 1.05 (0.60 , 1.84) | |
| **Model 2** | | | | | | |
| Never smoker | 1 | <.001 | 1 | .33 | 1 | .09 |
| Predominantly non-smoker | 1.80 (1.26 , 2.59) | | 1.08 (0.86 , 1.35) | | 1.16 (0.87 , 1.55) | |
| Predominantly smoker | 2.52 (1.65 , 3.84) | | 1.29 (0.97 , 1.71) | | 1.51 (1.06 , 2.17) | |
| Lifelong smoker | 2.11 (1.15 , 3.86) | | 0.94 (0.60 , 1.47) | | 1.13 (0.64 , 1.99) | |
| **Model 3** | | | | | | |
| Never smoker | 1 | <.001 | 1 | .46 | 1 | .17 |
| Predominantly non-smoker | 1.85 (1.28 , 2.66) | | 1.06 (0.84 , 1.33) | | 1.09 (0.82 , 1.47) | |
| Predominantly smoker | 2.43 (1.59 , 3.71) | | 1.22 (0.92 , 1.62) | | 1.37 (0.96 , 1.96) | |
| Lifelong smoker | 2.12 (1.15 , 3.91) | | 0.95 (0.60 , 1.48) | | 1.12 (0.63 , 1.99) | |
| **Model 4** | | | | | | |
| Never smoker | 1 | <.001 | 1 | .45 | 1 | .20 |
| Predominantly non-smoker | 1.69 (1.17 , 2.45) | | 1.05 (0.83 , 1.31) | | 1.12 (0.84 , 1.50) | |
| Predominantly smoker | 2.33 (1.52 , 3.58) | | 1.23 (0.93 , 1.64) | | 1.40 (0.98 , 2.00) | |
| Lifelong smoker | 2.08 (1.12 , 3.86) | | 0.93 (0.59 , 1.46) | | 1.03 (0.59 , 1.81) | |
| **Model 5** | | | | | | |
| Never smoker | 1 | <.001 | 1 | .37 | 1 | .20 |
| Predominantly non-smoker | 1.77 (1.23 , 2.55) | | 1.07 (0.86 , 1.34) | | 1.13 (0.85 , 1.51) | |
| Predominantly smoker | 2.47 (1.62 , 3.76) | | 1.27 (0.96 , 1.68) | | 1.40 (0.99 , 2.00) | |
| Lifelong smoker | 2.13 (1.16 , 3.91) | | 0.93 (0.60 , 1.46) | | 1.02 (0.58 , 1.80) | |
| **Model 6** | | | | | | |
| Never smoker | 1 | .002 | 1 | .90 | 1 | .15 |
| Predominantly non-smoker | 1.70 (1.16 , 2.49) | | 1.01 (0.80 , 1.28) | | 1.10 (0.82 , 1.48) | |
| Predominantly smoker | 2.10 (1.34 , 3.28) | | 1.12 (0.83 , 1.51) | | 1.39 (0.97 , 2.01) | |
| Lifelong smoker | 1.88 (0.99 , 3.57) | | 0.87 (0.54 , 1.38) | | 1.15 (0.64 , 2.06) | |

Note: RRR = relative risk ratio; CWP = chronic widespread pain; CRP = chronic regional pain

Model adjustments:

1: sex

2: sex and SEP (education at age 26, own occupational class)

3: sex and other health behaviours (BMI, alcohol intake and physical activity)

4: sex and longstanding illness or health problems

5: sex and symptoms of anxiety and depression (GHQ-28)

6: all covariates included in models 1 to 5



For peer review only - http://bmjopen.bmj.com/site/about/guidelines.xhtml

Supplementary information

Supplemental Table 1: Summary of smoking history data (i.e. those with at least 1 wave of smoking data and pain at age 68, n=2409)

| | Men, N (%) | Women, N (%) | P value |
|---|---|---|---|
| **Ever smoker, N=2409** | | | |
| No | 310 (26.8) | 431 (34.5) | <.001 |
| Yes | 848 (73.2) | 820 (65.6) | |
| | | | |
| **Smoking history at** | | | |
| **Age 20, n=2074** | | | |
| Current smoker | 551 (55.8) | 508 (46.8) | <.001 |
| Ex-smoker | 129 (13.1) | 145 (13.4) | |
| Never | 308 (31.2) | 433 (39.9) | |
| **Age 25, N=1956** | | | |
| Current smoker | 390 (42.4) | 344 (33.2) | <.001 |
| Ex-smoker | 255 (27.8) | 302 (29.1) | |
| Never | 274 (29.8) | 391 (37.7) | |
| **Age 31, N=2032** | | | |
| Current smoker | 375 (38.5) | 373 (35.3) | .001 |
| Ex-smoker | 336 (34.5) | 318 (30.1) | |
| Never | 263 (27) | 367 (34.7) | |
| **Age 36, N=2129** | | | |

For peer review only

| | Men, N (%) | Women, N (%) | P value |
|---|---|---|---|
| Current smoker | 301 (29.7) | 298 (26.8) | .001 |
| Ex-smoker | 431 (42.5) | 423 (38) | |
| Never | 283 (27.9) | 393 (35.3) | |
| **Age 43, N=2196** | | | |
| Current smoker | 278 (26.6) | 270 (23.5) | <.001 |
| Ex-smoker | 397 (38) | 312 (27.1) | |
| Never | 371 (35.5) | 568 (49.4) | |
| **Age 53, N=2157** | | | |
| Current smoker | 201 (19.9) | 210 (18.3) | <.001 |
| Ex-smoker | 437 (43.2) | 348 (30.4) | |
| Never | 374 (37.0) | 587 (51.3) | |
| **Age 60-64, N=2027** | | | |
| Current smoker | 109 (11.4) | 120 (11.2) | <.001 |
| Ex-smoker | 452 (47.3) | 367 (34.3) | |
| Never | 395 (41.3) | 584 (54.5) | |
| **Age 68, N=2366** | | | |
| Current smoker | 113 (9.9) | 96 (7.8) | <.001 |
| Ex-smoker | 537 (47.1) | 413 (33.7) | |
| Never | 489 (48.9) | 718 (58.5) | |



For peer review only - http://bmjopen.bmj.com/site/about/guidelines.xhtml

Supplemental Table 2: Association between lifetime smoking history (vs never smoker) and pain outcomes, n=1561 (complete case analysis)

| | **CWP vs no pain** | | **CRP vs no pain** | | **Other pain vs no pain** | |
|---|---|---|---|---|---|---|
| Model | RRR (95% CI) | P value | RRR (95% CI) | P value | RRR (95% CI) | P value |
| *Sex-adjusted* | | | | | | |
| Never smoker | 1 | .0012 | 1 | .13 | 1 | .21 |
| Predominantly non-smoker | 2.15 (1.40, 3.32) | | 1.25(0.95 , 1.64) | | 1.12 (0.77, 1.61) | |
| Predominantly smoker | 2.54 (1.52 , 4.23) | | 1.43 (1.02 , 2.01) | | 1.57 (1.01 , 2.44) | |
| Lifelong smoker | 1.52 (0.62 , 3.73) | | 0.89 (0.48 , 1.64) | | 0.99 (0.45, 2.17) | |
| | | | | | | |
| *Fully-adjusted** | | | | | | |
| Never smoker | 1 | .0064 | 1 | .24 | 1 | .27 |
| Predominantly non-smoker | 2.07 (1.31 , 3.25) | | 1.21 (0.91, 1.61) | | 1.08 (0.75, 1.57) | |
| Predominantly smoker | 2.33 (1.34 , 4.05) | | 1.33 (0.93, 1.92) | | 1.55 (0.98, 2.44) | |
| Lifelong smoker | 1.39 (0.53 , 3.60) | | 0.82 (0.43 , 1.55) | | 1.10 (0.49 , 2.48) | |

*Adjusted for all covariates (sex, own occupational class, educational level, BMI, leisure time physical activity, alcohol intake, longstanding illness and symptoms of anxiety and depression.)



For peer review only - http://bmjopen.bmj.com/site/about/guidelines.xhtml

For peer review only



For peer review only - http://bmjopen.bmj.com/site/about/guidelines.xhtml

Supplemental Table 3: Hazard ratio for all-cause mortality by lifetime smoking history

| | Sex adjusted | | Adjusted* | |
|---|---|---|---|---|
| Model | HR (95% CI) | P value | HR (95% CI) | P value |
| *Unadjusted* | | | | |
| Never smoker | 1 | | 1 | |
| Predominantly non-smoker | 1.15 (0.85 – 1.55) | .36 | 1.16 (0.86 – 1.56) | .33 |
| Predominantly smoker | 1.55 (1.12 – 2.15) | .008 | 1.49 (1.08 – 2.07) | .016 |
| Lifelong smoker | 5.16 (3.87 – 6.86) | <.001 | 4.76 (3.56 – 6.37) | <.001 |

*adjusted for sex, father's occupational class and own occupational class

For peer review only

**STROBE 2007 (v4) Statement—Checklist of items that should be included in reports of *cohort studies***

| Section/Topic | Item # | Recommendation | Reported on page # |
|---|---|---|---|
| **Title and abstract** | 1 | (*a*) Indicate the study's design with a commonly used term in the title or the abstract | 1, 2 |
| | | (*b*) Provide in the abstract an informative and balanced summary of what was done and what was found | 2, 3 |
| **Introduction** | | | |
| Background/rationale | 2 | Explain the scientific background and rationale for the investigation being reported | 4 |
| Objectives | 3 | State specific objectives, including any prespecified hypotheses | 5 |
| **Methods** | | | |
| Study design | 4 | Present key elements of study design early in the paper | 5 |
| Setting | 5 | Describe the setting, locations, and relevant dates, including periods of recruitment, exposure, follow-up, and data collection | 5,6 |
| Participants | 6 | (*a*) Give the eligibility criteria, and the sources and methods of selection of participants. Describe methods of follow-up | 5 |
| | | (*b*) For matched studies, give matching criteria and number of exposed and unexposed | |
| Variables | 7 | Clearly define all outcomes, exposures, predictors, potential confounders, and effect modifiers. Give diagnostic criteria, if applicable | 6-8 |
| Data sources/ measurement | 8* | For each variable of interest, give sources of data and details of methods of assessment (measurement). Describe comparability of assessment methods if there is more than one group | 8 |
| Bias | 9 | Describe any efforts to address potential sources of bias | 8,9 |
| Study size | 10 | Explain how the study size was arrived at | 9 |
| Quantitative variables | 11 | Explain how quantitative variables were handled in the analyses. If applicable, describe which groupings were chosen and why | 9, 10 |
| Statistical methods | 12 | (*a*) Describe all statistical methods, including those used to control for confounding | 9,10 |
| | | (*b*) Describe any methods used to examine subgroups and interactions | 9,10 |
| | | (*c*) Explain how missing data were addressed | 9,10 |
| | | (*d*) If applicable, explain how loss to follow-up was addressed | 10 |
| | | (*e*) Describe any sensitivity analyses | 10 |
| **Results** | | | |

For peer review only

| | | | |
|---|---|---|---|
| Participants | 13* | (a) Report numbers of individuals at each stage of study—eg numbers potentially eligible, examined for eligibility, confirmed eligible, included in the study, completing follow-up, and analysed | 5,9,10 |
| | | (b) Give reasons for non-participation at each stage | 5, 9, 10 |
| | | (c) Consider use of a flow diagram | |
| Descriptive data | 14* | (a) Give characteristics of study participants (eg demographic, clinical, social) and information on exposures and potential confounders | 9, 21 |
| | | (b) Indicate number of participants with missing data for each variable of interest | 8,9 |
| | | (c) Summarise follow-up time (eg, average and total amount) | 5,6 |
| Outcome data | 15* | Report numbers of outcome events or summary measures over time | 5,6 |
| Main results | 16 | (*a*) Give unadjusted estimates and, if applicable, confounder-adjusted estimates and their precision (eg, 95% confidence interval). Make clear which confounders were adjusted for and why they were included | 9,10, 23 |
| | | (*b*) Report category boundaries when continuous variables were categorized | |
| | | (*c*) If relevant, consider translating estimates of relative risk into absolute risk for a meaningful time period | |
| Other analyses | 17 | Report other analyses done—eg analyses of subgroups and interactions, and sensitivity analyses | 24,27 |
| **Discussion** | | | |
| Key results | 18 | Summarise key results with reference to study objectives | |
| **Limitations** | | | 10,11 |
| Interpretation | 20 | Give a cautious overall interpretation of results considering objectives, limitations, multiplicity of analyses, results from similar studies, and other relevant evidence | 11-13 |
| Generalisability | 21 | Discuss the generalisability (external validity) of the study results | 11-13 |
| **Other information** | | | |
| Funding | 22 | Give the source of funding and the role of the funders for the present study and, if applicable, for the original study on which the present article is based | 14 |

*Give information separately for cases and controls in case-control studies and, if applicable, for exposed and unexposed groups in cohort and cross-sectional studies.

**Note:** An Explanation and Elaboration article discusses each checklist item and gives methodological background and published examples of transparent reporting. The STROBE checklist is best used in conjunction with this article (freely available on the Web sites of PLoS Medicine at http://www.plosmedicine.org/, Annals of Internal Medicine at http://www.annals.org/, and Epidemiology at http://www.epidem.com/). Information on the STROBE Initiative is available at www.strobe-statement.org.

For peer review only

BMJ Open

# Lifetime cigarette smoking and chronic widespread and regional pain in later adulthood: Evidence from the 1946 British birth cohort study

| | |
|---|---|
| Journal: | *BMJ Open* |
| Manuscript ID | bmjopen-2018-021896.R1 |
| Article Type: | Research |
| Date Submitted by the Author: | 19-Apr-2018 |
| Complete List of Authors: | Bendayan, Rebecca; University College London, Population, Policy and Practice Programme<br>Cooper, Rachel; UCL, MRC Unit for Lifelong Health and Ageing at UCL<br>Muthuri, Stella; MRC Unit for Lifelong Health and Ageing, |
| <b>Primary Subject Heading</b>: | Epidemiology |
| Secondary Subject Heading: | Smoking and tobacco, Public health, Rheumatology |
| Keywords: | Chronic Pain, Cigarette Smoking, Life Course Epidemiology, Birth Cohort |
| | |

SCHOLARONE™
Manuscripts

**Lifetime cigarette smoking and chronic widespread and regional pain in later adulthood: Evidence from the 1946 British birth cohort study**

Rebecca Bendayan[1;2], Rachel Cooper[1], Stella G Muthuri[1]

[1] MRC Unit for Lifelong Health and Ageing at UCL, 33 Bedford Place, London, WC1B 5JU, UK

[2]Population, Policy and Practice Programme, UCL Institute of Child Health, 30 Guilford Street, London, WC1N 1EH UK, United Kingdom.

Correspondence to: Rebecca Bendayan, r.bendayan@ucl.ac.uk

The Corresponding Author has the right to grant on behalf of all authors and does grant on behalf of all authors, a worldwide licence to the Publishers and its licensees in perpetuity, in all forms, formats and media (whether known now or created in the future), to i) publish, reproduce, distribute, display and store the Contribution, ii) translate the Contribution into other languages, create adaptations, reprints, include within collections and create summaries, extracts and/or, abstracts of the Contribution, iii) create any other derivative work(s) based on the Contribution, iv) to exploit all subsidiary rights in the Contribution, v) the inclusion of electronic links from the Contribution to third party material where-ever it may be located; and, vi) licence any third party to do any or all of the above.

Key words: Chronic Pain; Cigarette Smoking; Life Course Epidemiology; Birth Cohort



For peer review only - http://bmjopen.bmj.com/site/about/guidelines.xhtml

**Abstract**

**Objective:** To examine whether different lifetime patterns of cigarette smoking are associated with chronic widespread and regional pain at age 68.

**Design:** Prospective cohort study

**Setting:** England, Scotland and Wales

**Participants:** Up to 2347 men and women from the Medical Research Council National Survey of Health and Development, who have been followed up since birth in 1946 and provided sufficient information on cigarette smoking across adulthood to be classified as never smoker, predominantly non-smoker, predominantly smoker or lifelong smoker; and pain assessment at age 68.

**Primary outcome measures:** Pain was self-reported at age 68, and chronic widespread pain (CWP) was defined according to American College of Rheumatology criteria. Participants who reported having pain for ≥3 months but who did not meet the CWP definition were classified as having chronic regional pain (CRP); those who reported pain which had lasted for < 3 months were classified as 'other' pain. No pain was the reference group.

**Results:** Findings from multinomial logistic regression models indicated that compared with never smokers, predominantly non-smokers, predominantly smokers and lifelong smokers all had an increased risk of CWP; Relative-Risk-Ratios = 1.70 [95%CI 1.16 – 2.49]; 2.10 [95%CI 1.34 – 3.28] and 1.88 [95%CI 0.99 – 3.57], respectively, after adjusting for sex, own occupational class, educational level, body mass index, leisure time physical activity, alcohol intake, longstanding illness and symptoms of anxiety and depression. No association was observed between smoking history and CRP or other pain.

**Conclusions:** These results suggest that exposure to cigarette smoking at any stage in adulthood was associated with higher risk of CWP in later adulthood; highlighting the ongoing importance of smoking prevention programmes. It also suggests that assessment of



For peer review only - http://bmjopen.bmj.com/site/about/guidelines.xhtml

lifetime smoking behaviour may be more useful in identifying those at greater risk of CWP in later life than assessment of current smoking status.

**Strengths and limitations of this study**

This study uses prospective data on smoking behaviour ascertained across adulthood (from age 20 to 68) providing us with the opportunity to characterise different lifetime patterns of smoking.

- This study distinguishes chronic widespread pain from regional pain and examines the risk of both.
- Chronic pain was assessed at a single time point so we were not able to establish temporality.
- Lifelong smokers were under-represented in the study sample as they had greater rates of all-cause mortality.
- Despite losses to follow up over the 69 years of the study, the sample has remained broadly representative of the national population of England, Scotland and Wales born at a similar time.

For peer review only



For peer review only - http://bmjopen.bmj.com/site/about/guidelines.xhtml

## Introduction

Chronic pain is highly prevalent in older populations and has major adverse impacts on quality of life, wellbeing and activity participation[1-4]. Identifying modifiable risk factors associated with pain may inform interventions to prevent and/or alleviate its consequences in later life. Cigarette smoking is one of many modifiable behavioural risk factors that may play a role in the development and progression of chronic pain. While this risk factor has already been widely studied[5,6] with most longitudinal studies reporting weak or null associations[7-10], the majority of these have been in populations younger than 65.

Moreover, even when studies have included older adults these have usually only considered a single assessment of smoking status (either at baseline or at the current or most recent time point). This information has then been used to classify individuals as current, former or never smokers, or as ever or never smokers. This is unlikely to adequately capture the variability in lifetime exposure to smoking and its associated health risks due to changes in smoking behaviour across adulthood. Distinguishing people with different lifetime smoking histories and investigating differences in their health prospects may be especially important in older adults; although the prevalence of smoking has steadily declined across all age groups over the last few decades, the baby boom generation who are currently reaching old age were at high risk of exposure to smoking earlier in adulthood[11]. In addition, they are likely to have been exposed to anti-smoking campaigns and comprehensive tobacco control policies early enough in adulthood to have potentially benefited from the subsequent reductions in risk of smoking related diseases linked to premature mortality[12]. While people with some history of smoking are therefore now more likely to reach old age than they would have been in previous generations, whether they reach old age with more chronic conditions and symptoms such as pain remains to be established.



For peer review only - http://bmjopen.bmj.com/site/about/guidelines.xhtml

Using data from the MRC National Survey of Health and Development (NSHD), which has assessed smoking status at multiple time points across adulthood, this paper aims to examine whether different lifetime patterns of cigarette smoking are associated with chronic widespread and regional pain at age 68.

## Methods

### Study population

The MRC NSHD is a socially stratified population sample of 5362 single, legitimate births that occurred in England, Wales and Scotland in one week of March 1946. Participants have been prospectively followed from birth, and participation rates have remained high[13-16]. At the 24th data collection, conducted between 2014 and 2015 when study members were aged 68-69, study participants were first asked to complete a postal questionnaire at age 68 before then being invited to have a home visit by a research nurse at age 69[15]. Of the 2816 people in the target sample living in England, Scotland and Wales, 2370 (84.2%) completed a postal questionnaire. In addition, a postal questionnaire was sent to 126 study members living abroad who remain in contact with the study of whom 83 (65.9%) returned a questionnaire. No attempt was made to contact the remaining 2420 study members: 957 (17.8%) had already died, 620 (11.6%) had previously withdrawn from the study, 448 (8.3%) had emigrated and were no longer in contact with the study, and 395 (7.4%) had been untraceable for more than five years.

Ethical approval for the data collection at age 68-69 was obtained from the Queen Square Research Ethics Committee (14/LO/1073) and the Scotland A Research Ethics Committee (14/SS/1009) and written informed consent was obtained from study members.



For peer review only - http://bmjopen.bmj.com/site/about/guidelines.xhtml

For peer review only

**Patient and Public involvement statement**

Participants have a lifelong association with NSHD. Over the 72 years of the study, the research team has increasingly involved participants, in line with changing norms about conducting cohort studies, starting at age 16 (in 1962) with the annual dissemination of study findings in birthday cards. Participants receive personal letters whenever they raise queries or provide additional comments, including suggestions for new topics to study. In the last ten years, the research team has increased the level of participant involvement through invitations to study events (65th and 70th birthday celebrations), public engagement activities, and focus groups to discuss clinical sub-studies. When piloting new questionnaires and assessments, we recruit patients from GP practices or from the UCLH Patient Public Involvement (PPI) group, and take into account their feedback when designing the mainstage fieldwork.

**Assessment of pain**

Information on pain was ascertained in the postal questionnaire administered at age 68. Participants were asked whether they had experienced any ache or pain in the last month which had lasted for one day or longer, not including pain occurring during the course of a feverish illness such as flu. Those who reported pain were then asked to report whether or not they had experienced this pain for at least 3 months and to shade the location of their pain using a four-view body manikin. Using American College of Rheumatology criteria[17] chronic widespread pain (CWP) was defined as pain present for 3 months or longer, both above and below the waist; on both the left and right side of the body; and in the axial skeleton. Participants who reported having pain for at least 3 months but who did not meet the CWP definition were classified as having chronic regional pain (CRP) and those who reported pain that had lasted for less than 3 months were classified as having 'other' pain[18].



For peer review only - http://bmjopen.bmj.com/site/about/guidelines.xhtml

### Assessment of smoking

Data on cigarette smoking were obtained on eight occasions across adulthood using postal questionnaires (at ages 20, 25, 31, 60-64, 68-69) and nurse interviews (at ages 36, 43, 53). At each of these ages, study members were asked whether they currently smoked cigarettes. Participants who provided a negative response were asked whether they had ever smoked. This information was used to construct a set of variables indicating smoking status at each age from 20 to 68, coded as current, former and never smoker. Using these 3-category variables, a lifetime smoking history variable was then constructed according to Clennell et al.[19]. Using this approach, we included those who provided smoking data for at least 3 waves and for whom missing data were not sequential at 3 or more waves (n=3705). Based on their reported smoking status at each age across adulthood, participants were grouped into one of four categories to summarise their smoking history: (1) participants who reported being non-smokers at all available data collections were classified as 'never smokers'; (2) participants who reported being smokers at all available data collections were classified as 'lifelong smokers'; participants who were classified as smokers at some ages and as ex or never smokers at other ages were classified as: (3) 'predominantly non-smoker' if they were a non-smoker on at least 3 occasions and more often reported being a non-smoker than a smoker; (4) 'predominantly smoker' if they reported being a smoker on 4 or more occasions and more often reported being a smoker than a non-smoker. For example, if a study member reported being a smoker on five occasions and an ex-smoker on two occasions, they were classified as 'predominantly smoker' whereas if they reported the reverse they were classified as 'predominantly non-smoker'[19].

### Covariates



For peer review only - http://bmjopen.bmj.com/site/about/guidelines.xhtml

Potential confounders were selected a priori based on previous research on chronic pain and health behaviours[20-23]. These were sex, socioeconomic position (indicated by own occupational class (which also allows us to distinguish between manual and non-manual work) and educational level), body mass index (BMI), other health behaviours (leisure time physical activity and alcohol intake), longstanding illness and symptoms of anxiety and depression. Unless otherwise specified these were assessed at age 68. Own occupation at age 53 years (or if not available, the most recent measure in adulthood) was categorised according to the Registrar General's social classification into three groups: high (I (Professional) or II (Managerial and technical)); middle (IIINM (skilled non-manual [NM]) or IIIM (skilled manual [M])); low (IV (partly skilled manual) or V (unskilled manual)). Highest educational level achieved by age 26 was categorised into three groups: no qualifications; up to O-level or equivalent; A level or equivalent and above. BMI ($kg/m^2$) was calculated from height (m) and weight (kg) measured by a trained nurse during the clinical assessment at age 69. Physical activity was assessed through self-reports of the level of participation in sports, vigorous leisure activities or exercise grouped as: inactive, moderately active (1-4 times/month) or most active (5 times or more/month). Similarly, alcohol intake in the last year was self-reported as never, only on special occasions, monthly or less, 2-4 times per month, 2-3 times per week, or 4 or more times per week. In addition, participants were asked whether they had any longstanding illness or health problems which had lasted, or were expected to last for 6 months or more. Symptoms of anxiety and depression were assessed using the 28-item General Health Questionnaire (GHQ-28). Each GHQ-28 item was coded using the General Scoring Method (i.e. 0 for response choices 1 and 2; and as 1 or response choices 3 and 4). Symptoms of anxiety and depression were indicated by summing responses to all items and applying a threshold for caseness of 5 or more[24].



For peer review only - http://bmjopen.bmj.com/site/about/guidelines.xhtml

## Statistical analyses

Multinomial logistic regression models were used to examine the associations between lifetime smoking history and pain outcomes (with no pain as the reference category). Models were first adjusted for sex and formal tests of interaction between sex and lifetime smoking history were undertaken. As no evidence of interactions with sex were found, subsequent models were sex adjusted. We then adjusted for each set of covariates in turn before adjusting for all covariates simultaneously in a final model. All models included the maximum number of participants with complete data on lifetime smoking history and pain (n=2347). To reduce data loss, covariates with missing values (i.e. educational attainment [n=114], occupational class [n=6], BMI [n=435], leisure time physical activity [n=25], alcohol intake [n=25], longstanding illness or health problems [n=24] and GHQ-28 [n=422] were imputed using multiple imputation by chained equations[25]. Analyses were performed across 20 imputed datasets and combined using Rubin's rules. In sensitivity analyses, models were rerun on the sample with complete data on all covariates (n=1759). All analyses were conducted using STATA v14.1.

## Results

Of the total analytical sample of 2347, 1304 (55.6%) reported pain, with 10.6% reporting CWP, 30.3% reporting CRP, and 14.7% reporting 'other pain'. More women than men reported CWP (13.2% for women vs 7.7% for men, p<.001). Overall, the prevalence of smoking declined across adulthood from 55.8% for men and 46.8% of women at age 20 to 9.9% for men and 7.8% for women by age 68 (p-values for sex differences at both ages<.001) (Supplementary Table 1). The prevalence of lifelong non-smokers was 34.3% for women and 26.3% for men. Of those classified as predominantly non-smokers, 61.6% had stopped



For peer review only - http://bmjopen.bmj.com/site/about/guidelines.xhtml

smoking at least once by age 25 and only 0.5% were smokers at the most recent report. Of those classified as predominantly smokers, 88.9% were smokers at age 25, but only 14.2% were still smokers at the most recent report. Descriptive statistics are shown in Table 1.

PLEASE, INSERT TABLE 1 AROUND HERE

In sex-adjusted models, predominantly non-smokers, predominantly smokers, and lifelong smokers all had a higher likelihood of CWP, compared with lifelong non-smokers (Relative-Risk Ratios (RRR) of CWP vs no pain=1.80 (95%CI 1.26 – 2.58), 2.64 (95%CI 1.74 – 3.99) and 2.27 (95%CI 1.25 – 4.13), respectively (Table 2, Model 1). Adjustment for each set of covariates had only limited impact and associations were maintained in fully-adjusted models; RRRs = 1.70 (95%CI 1.16 – 2.49) for predominantly non-smokers, 2.10 (95%CI 1.34 – 3.28) for predominantly smokers and 1.88 (95%CI 0.99 – 3.57) for lifelong smokers (Table 2, Model 6). No associations were observed between lifetime smoking history and CRP or other pain (Table 2).

PLEASE, INSERT TABLE 1 AROUND HERE

When analyses were restricted to those with complete data (n=1759), we found similar results and the overall conclusions remained the same (Supplemental Table 2).

## Discussion

The main aim of this study was to examine whether different patterns of cigarette smoking over the life course were associated with CWP and CRP at age 68 in a nationally representative birth cohort. Our results showed that exposure to cigarette smoking at any time point across adulthood was associated with increased risk of CWP at age 68, even after adjustment for a range of potential confounders and mediators. However, there was no evidence of associations between lifetime cigarette smoking and CRP or other pain.



For peer review only - http://bmjopen.bmj.com/site/about/guidelines.xhtml

*Comparisons with other studies*

Our finding of an association between different patterns of lifetime smoking and CWP in older adults builds on findings from other previous studies of chronic pain in older adults. For example, in a longitudinal study of British adults aged 50 years or older, individuals who were current smokers had higher odds of pain at baseline but there was no evidence for increased likelihood of new-onset widespread pain over 3 years of follow-up when former and current smokers were combined in one group and compared with never smokers[23]. Likewise, a cross-sectional study found that smoking status was only associated with chronic pain prevalence when current and former smokers were combined in one group and compared with never smokers among people aged 65 years and older in Sweden[26]. By using data collected prospectively across adulthood we were able to distinguish different lifetime patterns of cigarette smoking and go beyond previous research. This has strengths when compared with other studies that have relied on single assessments of smoking and may therefore not adequately or reliably capture all relevant lifetime exposure.

*Potential explanations of findings*

Our finding of increased risk of CWP among all smoking groups (i.e. predominantly non-smokers, predominantly smokers and lifelong smokers), when compared with never smokers suggests that cigarette smoking even for a short period of time over the life course may have a long-lasting impact on the probability of reporting CWP in later adulthood. One plausible explanation for this finding is that smoking may produce changes in central nervous system function that increase susceptibility to pain the effects of which continue to persist after smoking cessation[27]. Another consideration is that the associations between smoking and pain may be bi-directional; not only is there evidence that tobacco smoking may be a risk factor for the development and exacerbation of pain but also that pain may serve to motivate



For peer review only - http://bmjopen.bmj.com/site/about/guidelines.xhtml

smoking[7]. For this reason, it is possible that some individuals in our study may have developed CWP in early adulthood and used smoking as a coping strategy for this with this then causing them to experience greater difficulty quitting[28].

That associations were observed between lifetime smoking and CWP but not with CRP or other pain suggests that any influence of smoking on pain is likely to be operating via mechanisms related specifically to the development of CWP. This is consistent with the idea that CWP and CRP may have different underlying aetiologies[7]. For example, some studies have suggested that nicotine may alter pain regulatory mechanisms (e.g. potential alteration of pain processing or cause structural changes to other systems) which might have long-term consequences for painful chronic conditions[29]. The associations between lifetime smoking and CWP were only partially attenuated after adjustment for a range of factors including sex, socioeconomic position, body size, other health behaviour, longstanding illness and symptoms of anxiety and depression. This suggests that there might be other relevant explanatory variables. For example, although affective symptoms have been taken into consideration in our study some psychosocial models have highlighted the role of pain-related stress, fear or anxiety sensitivity in the transition from acute to chronic pain[7,30,31]. These pain specific measures were not available in our data and therefore future studies are encouraged to include these and examine their association with CWP onset and smoking.

*Methodological considerations*

One of the main strengths of our study was the use of data from a large nationally representative sample of older adults with prospective ascertainment of smoking status across adulthood which allowed us to characterise different lifetime patterns of smoking. Although we were unable to validate self-reports of smoking using cotinine measurements or reliably distinguish between light and heavy smokers, our approach does still have important strengths when compared with the commonly used categorisation of never, ex or current



For peer review only - http://bmjopen.bmj.com/site/about/guidelines.xhtml

smoker based on self-reported smoking at a single time point. While the use of recalled information on quitting attempts between assessments could have introduced bias, when we performed sensitivity analyses to check for potential inconsistencies in reporting and compared results with retrospective measures on time since quitting for former smokers there were no differences in findings. However, the observed increase in prevalence of never smokers at older ages (Supplementary table 2) suggests that smokers were more likely than non-smokers to be lost to follow-up. This is partly explained by the fact that lifelong smokers had much greater rates of all-cause mortality across adulthood up to age 69 (Supplementary table 3); survival bias may therefore explain why predominantly smokers were found to have higher relative risks of CWP than lifelong smokers when both groups were compared with never smokers.

Another potential limitation of our analyses is that we cannot rule out reverse causation as chronic pain was only assessed at one time point. As noted above, recent research has highlighted that the association between smoking and pain is likely to be bi-directional[28,32] and so future longitudinal studies with prospective ascertainment of both smoking and pain across life should be performed. Such studies would also benefit from assessment of pain severity and information on potential causes of pain, such as history of accident, which we were not able to take account of in our analyses but which may provide important additional insights. Although our findings are generalizable to the current generation of adults reaching old age in the UK, changing trends in smoking behaviour over the last 70 years[11,33], should be considered and further research is needed to examine whether similar associations will be observed in more recently born cohorts who will have experienced different selection effects into smoking and different lifetime patterns of exposure to smoking.



For peer review only - http://bmjopen.bmj.com/site/about/guidelines.xhtml

*Conclusions*

Our results highlight the role of smoking at any time point across adulthood as an independent risk factor for CWP in later adulthood, after considering socio-economic, psychosocial and health-related factors. The evidence provided for the long-lasting effect of smoking on this condition highlights the potential benefit of considering smoking history rather than current smoking status when identifying those at greater risk of CWP in later life especially as opportunities to prevent CWP may be missed if only current smokers are classified as higher risk. These findings are relevant from a clinical and public health perspective, as smoking is a modifiable risk factor and our findings suggest that its primary prevention may be key.

For peer review only

**Acknowledgements:** The authors are grateful to NSHD study members for their continuing support. We also thank members of the NSHD scientific and data collection teams who have been involved in NSHD data collections.

**Funding:** This work was supported by the UK Medical Research Council who provide support for RC and SM (Programme code MC_UU_12019/4) and fund the MRC National Survey of Health and Development. RB was supported by a grant from the Spanish Ministry of Science and Innovation (PSI2013-42512- P) The funders of the study had no role in the study design, data collection, data analysis, data interpretation, writing of the report or the decision to submit the article for publication.

**Open access:** This is an Open Access article distributed in accordance with the terms of the Creative Commons Attribution (CC BY 3.0) license, which permits others to distribute, remix, adapt and build upon this work, for commercial use, provided the original work is properly cited. See: http://creativecommons.org/licenses/by/3.0.

**Declaration of competing interests:** All authors have completed the ICMJE uniform disclosure form at www.icmje.org/coi_disclosure.pdf and declare: RC and SM received financial support from the UK Medical Research Council for the submitted work; no financial relationships with any organisations that might have an interest in the submitted work in the previous three years; no other relationships or activities that could appear to have influenced the submitted work.

**Author contributions:** RB and SGM conceived the idea for this study and along with RC developed the study objectives. RC was part of the team responsible for the 68-69 year data collection. RB and SGM undertook statistical analyses. All authors contributed to the interpretation of the data. RB and SGM drafted the article and all authors contributed to its critical revision and provided final approval of the version to be published. RB and SGM are



For peer review only - http://bmjopen.bmj.com/site/about/guidelines.xhtml

the guarantors and accept full responsibility for the work and the conduct of the study, had access to the data, and controlled the decision to publish. All authors had full access to all of the data (including statistical reports and tables) in the study and can take responsibility for the integrity of the data and the accuracy of the data analysis.

**Transparency declaration:** RB and SGM affirm that the manuscript is an honest, accurate, and transparent account of the study being reported and that no important aspects of the study have been omitted.

**Data sharing statement:** Data are available on request to the NSHD Data Sharing Committee. NSHD data sharing policies and processes meet the requirements and expectations of MRC policy on sharing of data from population and patient cohorts: http://www.mrc.ac.uk/research/research-policy-ethics/data-sharing/policy/. Data requests should be submitted to mrclha.swiftinfo@ucl.ac.uk ; further details can be found at http://www.nshd.mrc.ac.uk/data.aspx DOI: 10.5522/NSHD/Q101;10.5522/NSHD/Q102; 10.5522/NSHD/Q103.

These policies and processes are in place to ensure that the use of data from this 71 year old national birth cohort study is within the bounds of consent given previously by study members, complies with MRC guidance on ethics and research governance, and meets rigorous MRC data security standards.



For peer review only - http://bmjopen.bmj.com/site/about/guidelines.xhtml

**References:**


1. Gardener EA, Huppert FA, Guralnik JM, Melzer D. Middle‐Aged and Mobility‐Limited: Prevalence of Disability and Symptom Attributions in a National Survey. *J Gen Intern Med* 2006;*21*(10):1091-1096. doi:10.1111/j.1525-1497.2006.00564.x
2. Leveille SG, Bean J, Ngo L, *et al.* (2007). The pathway from musculoskeletal pain to mobility difficulty in older disabled women. *Pain* 2007;*128*(1): 69-77. doi:10.1016/j.pain.2006.08.031
3. Leveille SG, Fried L, Guralnik JM. Disabling symptoms. *J Gen Intern Med* 2002;*17*(10):766-773. doi:10.1046/j.1525-1497.2002.20229.x
4. Patel KV, Guralnik JM, Dansie EJ, Turk DC. Prevalence and impact of pain among older adults in the United States: findings from the 2011 National Health and Aging Trends Study. *Pain* 2013;*154*(12):2649-2657. doi:10.1016/j.pain.2013.07.029
5. Ditre JW, Brandon TH, Zale EL, Meagher MM. Pain, nicotine, and smoking: research findings and mechanistic considerations. *Psychol Bull* 2011;*137*(6):1065. doi:10.1037/a0025544
6. Parkerson HA, Zvolensky MJ, Asmundson GJ. Understanding the relationship between smoking and pain. *Expert Rev Neurother* 2013;*13*(12):1407-1414. doi:10.1586/14737175.2013.859524
7. Shiri R, Karppinen J, Leino-Arjas P, *et al.* The Association between Smoking and Low Back Pain: A Meta-analysis. *Am J Med* 2010;*123*(1):87.e7-87.e35. doi:10.1016/j.amjmed.2009.05.028
8. Mundal I, Gråwe R, Bjørngaard J, *et al.* Prevalence and long-term predictors of persistent chronic widespread pain in the general population in an 11-year prospective study: the HUNT study. *BMC Musculoskelet Disord* 2014, 15(1), 213. doi:10.1186/1471-2474-15-213




For peer review only - http://bmjopen.bmj.com/site/about/guidelines.xhtml

9. Volkman J, DeRicke EC, Driscoll MA, *et al.* Smoking status and pain intensity among OEF/OIF/OND veterans. *Pain Medicine* 2015;16(9):1690-1696. doi:10.1111/pme.12753

10. John U, Hanke M, Meyer, C, Volzke, H, Baumeister S, Alte D. Tobacco smoking in relation to pain in a national general population survey. *Prev Med* 2006;43(6): 477-481. doi: 10.1016/j.ypmed.2006.07.005

11. Davy M. Time and generational trends in smoking among men and women in Great Britain, 1972–2004/05. *Health Statistics Quarterly* 2006;*32*:35-43.

12. Pirie K, Peto R, Reeves GK, *et al.* The 21st century hazards of smoking and benefits of stopping: a prospective study of one million women in the UK. *Lancet* 2013;*381*(9861):133-141. doi:10.1016/S0140-6736(12)61720-6

13. Wadsworth M, Kuh D, Richards M, Hardy R. Cohort Profile: The 1946 National Birth Cohort (MRC National Survey of Health and Development). *Int J Epidemiol* 2006;35(1):49-54. doi:10.1093/ije/dyi201

14. Kuh D, Pierce M, Adams J, *et al.* Cohort profile: updating the cohort profile for the MRC National Survey of Health and Development: a new clinic-based data collection for ageing research. *Int J Epidemiol* 2011;40(1):e1-9. doi:10.1093/ije/dyq231

15. Kuh D, Wong A, Shah I, *et al.* The MRC National Survey of Health and Development reaches age 70: maintaining participation at older ages in a birth cohort study. *Eur J Epidemiol* 2016;31(11):1135-1147. doi:10.1007/s10654-016-0217-8

16. Stafford M, Black S, Shah I, *et al.* Using a birth cohort to study ageing: representativeness and response rates in the National Survey of Health and Development. *Eur J Ageing* 2013;*10*(2):145-157. doi:10.1007/s10433-013-0258-8

17. Wolfe F, Smythe HA, Yunus MB, *et al.* The American College of Rheumatology 1990 criteria for the classification of fibromyalgia. *Arthritis & Rheumatology* 1990;*33*(2): 160-172. doi:10.1002/art.1780330203



For peer review only - http://bmjopen.bmj.com/site/about/guidelines.xhtml

18. Muthuri SG, Kuh D, Bendayan R, *et al*. Chronic physical illness in early life and risk of chronic widespread and regional pain at age 68: evidence from the 1946 British birth cohort. *Pain* 2016;*157*(10):2382. doi:10.1097/j.pain.0000000000000663

19. Clennell S, Kuh D, Guralnik J, *et al*. Characterisation of smoking behaviour across the life course and its impact on decline in lung function and all-cause mortality: evidence from a British birth cohort. *J Epidemiol Community Health* 2008;*62*(12):1051-1056. doi:10.1136/jech.2007.068312

20. Bergman S. Psychosocial aspects of chronic widespread pain and fibromyalgia. *Disability and Rehabilitation* 2005;*27*(12):675-683. doi:10.1080/09638280400009030

21. Ferreira PH, Beckenkamp P, Maher CG, *et al.* (2013). Nature or nurture in low back pain? Results of a systematic review of studies based on twin samples. *Eur J Pain* 2013;*17*(7):957-971. doi:10.1002/j.1532-2149.2012.00277.x

22. Junqueira DRG, Ferreira ML, Refshauge K, *et al.* Heritability and lifestyle factors in chronic low back pain: results of the Australian Twin Low Back Pain Study (The AUTBACK study). *Eur J Pain* 2014;*18*(10):1410-1418. doi:10.1002/ejp.506

23. McBeth J, Lacey RJ, Wilkie R. Predictors of New□Onset Widespread Pain in Older Adults: Results From a Population□Based Prospective Cohort Study in the UK. *Arthritis & Rheumatology* 2014;*66*(3):757-767. doi:10.1002/art.38284

24. Goldberg DP, Hillier VF. A scaled version of the General Health Questionnaire. *Psychol Med* 1979;9(1):139-145. doi:10.1017/S0033291700021644

25. Sterne JAC, White IR, Carlin JB, *et al.* Multiple imputation for missing data in epidemiological and clinical research: potential and pitfalls. *BMJ* 2009;338.

26. Jakobsson U, Larsson C. Smoking and chronic pain among people aged 65 years and older. *Pain Practice* 2014;*14*(3):237-244. doi:10.1111/papr.12067



For peer review only - http://bmjopen.bmj.com/site/about/guidelines.xhtml

27. Perkins KA, Grobe JE, Stiller RL, *et al.* Effects of nicotine on thermal pain detection in humans. *Exp Clin Psychopharmacol*;2:95-106.

28. Ditre JW, Kosiba JD, Zale EL, *et al.* Chronic pain status, nicotine withdrawal, and expectancies for smoking cessation among lighter smokers. *Ann Behav Med* 2016;*50*(3):427-435. doi:10.1007/s12160-016-9769-9

29. Shi Y, Weingarten TN, Mantilla CB, *et al.* Smoking and Pain Pathophysiology and Clinical Implications. *The Journal of the American Society of Anesthesiologists* 2010;*113*(4): 977-992.

30. Asmundson GJ, Norton PJ, Vlaeyen JW. Fear-avoidance models of chronic pain: an overview. *Understanding and Treating Fear of Pain* 2004;3-24.

31. Vlaeyen JW, Linton SJ. Fear-avoidance and its consequences in chronic musculoskeletal pain: a state of the art. *Pain* 2000;*85*(3):317-332. doi:10.1016/S0304-3959(99)00242-0

32. Zale EL, Maisto SA, Ditre JW. Anxiety and depression in bidirectional relations between pain and smoking: Implications for smoking cessation. *Behavior Modification* 2016;*40*(1-2):7-28. doi:10.1177/0145445515610744

33. Thun MJ, Carter BD, Feskanich D, et al. 50-year trends in smoking-related mortality in the United States. *N Engl J Med* 2013;*368*(4):351-364. doi:10.1056/NEJMsa1211127

For peer review only



For peer review only - http://bmjopen.bmj.com/site/about/guidelines.xhtml

Table 1: Characteristics of participants from the MRC National Survey of Health and Development by sex at age 68 [Maximum N=2347]

| | Men, N† (%) | Women, N† (%) | P value* |
|---|---|---|---|
| Total | 1,123 (47.9) | 1,224 ( 52.1) | |
| **Pain at age 68** | | | |
| No pain | 537 (47.8) | 506 (41.3) | <.001 |
| Other pain | 184 (16.4) | 162 (13.2) | |
| Chronic Regional Pain | 316 (28.1) | 394 (32.2) | |
| Chronic Widespread Pain | 86 (7.7) | 162 (13.2) | |
| **Lifetime smoking history** | | | |
| Never smoker | 295 (26.3) | 420 (34.3) | <.001 |
| Predominantly non-smoker | 527 (46.9) | 516 (42.2) | |
| Predominantly smoker | 228 (20.3) | 224 (18.3) | |
| Lifelong smoker | 73 (6.5) | 64 (5.2) | |
| **BMI (kg/m$^2$) at age 69** (n=1919) | | | |
| Mean (SD) | 28.0 (4.46) | 28.1 (5.56) | .92 |
| **Occupational class at age 53** | | | |
| High (I/II) | 631 (56.3) | 458 (37.5) | <.001 |
| Middle (IIINM/ IIIM) | 385 (34.4) | 543 (44.5) | |
| Low (IV/V) | 104 (9.3) | 220 (18.0) | |
| **Educational attainment by age 26** | | | |
| A level or equivalent & above | 505 (47.2) | 374 (32.2) | <.001 |
| Up to O level or equivalent | 212 (19.8) | 419 (36.1) | |
| None | 354 (33.1) | 369 (31.8) | |
| **Alcohol intake at age 68** | | | |
| Never | 205 (18.5) | 396 (32.7) | <.001 |
| Monthly or less | 55 (5.0) | 107 (8.8) | |
| 2-4 times per month | 157 (14.1) | 203 (16.8) | |
| 2-3 times per week | 295 (26.6) | 262 (21.6) | |
| ≥4 times per week | 398 (35.9) | 244 (20.1) | |
| **Participation in leisure time physical activity at age 68** | | | |
| Most active( ≥5 times/ month) | 319 (28.7) | 320 (26.4) | .18 |
| Moderately active ( 1-4 times/ month) | 123 (11.1) | 161 (13.3) | |
| Inactive | 669 (60.2) | 730 (60.3) | |
| **Longstanding illness or health problem at age 68** | | | |



For peer review only - http://bmjopen.bmj.com/site/about/guidelines.xhtml

| | Men, N[†] (%) | Women, N[†] (%) | P value[*] |
|---|---|---|---|
| No | 449 (40.3) | 508(42.0) | .38 |
| Yes | 666 (59.7) | 700 (57.9) | |
| **Symptoms of anxiety and depression (GHQ-28) at age 68** | | | |
| No (≤4) | 840 (90.7) | 829 (83.0) | <.001 |
| Yes (>4) | 86 (9.3) | 170 (17.0) | |

*comparison of sexes using student t-test or chi-square tests as appropriate

† Sample restricted to those participants with data on smoking history and pain. N varies due to missing data on covariates

For peer review only

Table 2. Associations between lifetime smoking history and pain at age 68 (n=2347)

| | CWP vs no pain | | CRP vs no pain | | Other pain vs no pain | |
|---|---|---|---|---|---|---|
| | RRR (95% CI) | P | RRR (95% CI) | P | RRR (95% CI) | P |
| **Model 1** | | | | | | |
| Never smoker | 1 | .0001 | 1 | .25 | 1 | .25 |
| Predominantly non-smoker | 1.80 (1.26 , 2.58) | | 1.08 (0.86 , 1.35) | | 1.14 (0.85 , 1.52) | |
| Predominantly smoker | 2.64 (1.74 , 3.99) | | 1.32 (0.99 , 1.74) | | 1.44 (1.01 , 2.04) | |
| Lifelong smoker | 2.27 (1.25 , 4.13) | | 0.97 (0.62 , 1.51) | | 1.05 (0.60 , 1.84) | |
| **Model 2** | | | | | | |
| Never smoker | 1 | <.001 | 1 | .33 | 1 | .09 |
| Predominantly non-smoker | 1.80 (1.26 , 2.59) | | 1.08 (0.86 , 1.35) | | 1.16 (0.87 , 1.55) | |
| Predominantly smoker | 2.52 (1.65 , 3.84) | | 1.29 (0.97 , 1.71) | | 1.51 (1.06 , 2.17) | |
| Lifelong smoker | 2.11 (1.15 , 3.86) | | 0.94 (0.60 , 1.47) | | 1.13 (0.64 , 1.99) | |
| **Model 3** | | | | | | |
| Never smoker | 1 | <.001 | 1 | .46 | 1 | .17 |
| Predominantly non-smoker | 1.85 (1.28 , 2.66) | | 1.06 (0.84 , 1.33) | | 1.09 (0.82 , 1.47) | |
| Predominantly smoker | 2.43 (1.59 , 3.71) | | 1.22 (0.92 , 1.62) | | 1.37 (0.96 , 1.96) | |
| Lifelong smoker | 2.12 (1.15 , 3.91) | | 0.95 (0.60 , 1.48) | | 1.12 (0.63 , 1.99) | |
| **Model 4** | | | | | | |
| Never smoker | 1 | <.001 | 1 | .45 | 1 | .20 |
| Predominantly non-smoker | 1.69 (1.17 , 2.45) | | 1.05 (0.83 , 1.31) | | 1.12 (0.84 , 1.50) | |
| Predominantly smoker | 2.33 (1.52 , 3.58) | | 1.23 (0.93 , 1.64) | | 1.40 (0.98 , 2.00) | |
| Lifelong smoker | 2.08 (1.12 , 3.86) | | 0.93 (0.59 , 1.46) | | 1.03 (0.59 , 1.81) | |
| **Model 5** | | | | | | |
| Never smoker | 1 | <.001 | 1 | .37 | 1 | .20 |
| Predominantly non-smoker | 1.77 (1.23 , 2.55) | | 1.07 (0.86 , 1.34) | | 1.13 (0.85 , 1.51) | |
| Predominantly smoker | 2.47 (1.62 , 3.76) | | 1.27 (0.96 , 1.68) | | 1.40 (0.99 , 2.00) | |
| Lifelong smoker | 2.13 (1.16 , 3.91) | | 0.93 (0.60 , 1.46) | | 1.02 (0.58 , 1.80) | |
| **Model 6** | | | | | | |
| Never smoker | 1 | .002 | 1 | .90 | 1 | .15 |
| Predominantly non-smoker | 1.70 (1.16 , 2.49) | | 1.01 (0.80 , 1.28) | | 1.10 (0.82 , 1.48) | |
| Predominantly smoker | 2.10 (1.34 , 3.28) | | 1.12 (0.83 , 1.51) | | 1.39 (0.97 , 2.01) | |
| Lifelong smoker | 1.88 (0.99 , 3.57) | | 0.87 (0.54 , 1.38) | | 1.15 (0.64 , 2.06) | |

Note: RRR = relative risk ratio; CWP = chronic widespread pain; CRP = chronic regional pain

Model adjustments:

1: sex

2: sex and SEP (education at age 26, own occupational class)

3: sex and other health behaviours (BMI, alcohol intake and physical activity)

4: sex and longstanding illness or health problems

5: sex and symptoms of anxiety and depression (GHQ-28)

6: all covariates included in models 1 to 5



For peer review only - http://bmjopen.bmj.com/site/about/guidelines.xhtml

Supplementary information

Supplementary Table 1: Summary of smoking history data (i.e. those with at least 1 wave of smoking data and pain at age 68, n=2409)

| | Men, N (%) | Women, N (%) | P value |
|---|---|---|---|
| **Ever smoker, N=2409** | | | |
| No | 310 (26.8) | 431 (34.5) | <.001 |
| Yes | 848 (73.2) | 820 (65.6) | |
| **Smoking history at** | | | |
| **Age 20, n=2074** | | | |
| Current smoker | 551 (55.8) | 508 (46.8) | <.001 |
| Ex-smoker | 129 (13.1) | 145 (13.4) | |
| Never | 308 (31.2) | 433 (39.9) | |
| **Age 25, N=1956** | | | |
| Current smoker | 390 (42.4) | 344 (33.2) | <.001 |
| Ex-smoker | 255 (27.8) | 302 (29.1) | |
| Never | 274 (29.8) | 391 (37.7) | |
| **Age 31, N=2032** | | | |
| Current smoker | 375 (38.5) | 373 (35.3) | .001 |
| Ex-smoker | 336 (34.5) | 318 (30.1) | |
| Never | 263 (27) | 367 (34.7) | |
| **Age 36, N=2129** | | | |
| Current smoker | 301 (29.7) | 298 (26.8) | .001 |

For peer review only

| | Men, N (%) | Women, N (%) | P value |
|---|---|---|---|
| Ex-smoker | 431 (42.5) | 423 (38) | |
| Never | 283 (27.9) | 393 (35.3) | |
| **Age 43, N=2196** | | | |
| Current smoker | 278 (26.6) | 270 (23.5) | <.001 |
| Ex-smoker | 397 (38) | 312 (27.1) | |
| Never | 371 (35.5) | 568 (49.4) | |
| **Age 53, N=2157** | | | |
| Current smoker | 201 (19.9) | 210 (18.3) | <.001 |
| Ex-smoker | 437 (43.2) | 348 (30.4) | |
| Never | 374 (37.0) | 587 (51.3) | |
| **Age 60-64, N=2027** | | | |
| Current smoker | 109 (11.4) | 120 (11.2) | <.001 |
| Ex-smoker | 452 (47.3) | 367 (34.3) | |
| Never | 395 (41.3) | 584 (54.5) | |
| **Age 68, N=2366** | | | |
| Current smoker | 113 (9.9) | 96 (7.8) | <.001 |
| Ex-smoker | 537 (47.1) | 413 (33.7) | |
| Never | 489 (48.9) | 718 (58.5) | |



For peer review only - http://bmjopen.bmj.com/site/about/guidelines.xhtml

Supplementary Table 2: Associations between lifetime smoking history (vs never smoker) and pain outcomes, n=1759 (complete case analysis)

| | **CWP vs no pain** | | **CRP vs no pain** | | **Other pain vs no pain** | |
|---|---|---|---|---|---|---|
| Model | RRR (95% CI) | P value | RRR (95% CI) | P value | RRR (95% CI) | P value |
| *Sex-adjusted* | | | | | | |
| Never smoker | 1 | .0028 | 1 | .17 | 1 | .18 |
| Predominantly non-smoker | 1.83 (1.22, 2.75) | | 1.14 (0.89 , 1.48) | | 1.11 (0.79, 1.57) | |
| Predominantly smoker | 2.39 (1.49 , 3.84) | | 1.34 (0.97 , 1.84) | | 1.53 (1.02 , 2.29) | |
| Lifelong smoker | 1.60 (0.75 , 3.46) | | 0.78 (0.45 , 1.37) | | 0.91 (0.45, 1.85) | |
| *Fully-adjusted** | | | | | | |
| Never smoker | 1 | .024 | 1 | .35 | 1 | .23 |
| Predominantly non-smoker | 1.76 (1.15 , 2.70) | | 1.10 (0.84, 1.44) | | 1.09 (0.77, 1.55) | |
| Predominantly smoker | 2.09 (1.26 , 3.47) | | 1.20 (0.86, 1.69) | | 1.52 (0.99, 2.31) | |
| Lifelong smoker | 1.47 (0.65 , 3.31) | | 0.73 (0.41 , 1.31) | | 0.99 (0.48 , 2.06) | |

*Adjusted for all covariates (sex, own occupational class, educational level, BMI, leisure time physical activity, alcohol intake, longstanding illness and symptoms of anxiety and depression.)



For peer review only - http://bmjopen.bmj.com/site/about/guidelines.xhtml

Supplementary Table 3: Hazard ratio for all-cause mortality by lifetime smoking history

| | Sex adjusted | | Adjusted* | |
|---|---|---|---|---|
| | HR (95% CI) | P value | HR (95% CI) | P value |
| Never smoker | 1 | | 1 | |
| Predominantly non-smoker | 1.21 (0.89 1.64) | .22 | 1.22 (0.90 – 1.65) | .21 |
| Predominantly smoker | 1.59 (1.14 – 2.22) | .007 | 1.52 (1.09 – 2.13) | .014 |
| Lifelong smoker | 5.12 (3.79 – 6.90) | <.001 | 4.71 (3.48 – 6.39) | <.001 |

*adjusted for sex, father's occupational class and own occupational class

For peer review only

**STROBE 2007 (v4) Statement—Checklist of items that should be included in reports of *cohort studies***

| Section/Topic | Item # | Recommendation | Reported on page # |
|---|---|---|---|
| **Title and abstract** | 1 | (*a*) Indicate the study's design with a commonly used term in the title or the abstract | 1, 2 |
| | | (*b*) Provide in the abstract an informative and balanced summary of what was done and what was found | 2, 3 |
| **Introduction** | | | |
| Background/rationale | 2 | Explain the scientific background and rationale for the investigation being reported | 4 |
| Objectives | 3 | State specific objectives, including any prespecified hypotheses | 5 |
| **Methods** | | | |
| Study design | 4 | Present key elements of study design early in the paper | 5 |
| Setting | 5 | Describe the setting, locations, and relevant dates, including periods of recruitment, exposure, follow-up, and data collection | 5,6 |
| Participants | 6 | (*a*) Give the eligibility criteria, and the sources and methods of selection of participants. Describe methods of follow-up | 5 |
| | | (*b*) For matched studies, give matching criteria and number of exposed and unexposed | |
| Variables | 7 | Clearly define all outcomes, exposures, predictors, potential confounders, and effect modifiers. Give diagnostic criteria, if applicable | 6-8 |
| Data sources/ measurement | 8* | For each variable of interest, give sources of data and details of methods of assessment (measurement). Describe comparability of assessment methods if there is more than one group | 8 |
| Bias | 9 | Describe any efforts to address potential sources of bias | 8,9 |
| Study size | 10 | Explain how the study size was arrived at | 9 |
| Quantitative variables | 11 | Explain how quantitative variables were handled in the analyses. If applicable, describe which groupings were chosen and why | 9, 10 |
| Statistical methods | 12 | (*a*) Describe all statistical methods, including those used to control for confounding | 9,10 |
| | | (*b*) Describe any methods used to examine subgroups and interactions | 9,10 |
| | | (*c*) Explain how missing data were addressed | 9,10 |
| | | (*d*) If applicable, explain how loss to follow-up was addressed | 10 |
| | | (*e*) Describe any sensitivity analyses | 10 |
| **Results** | | | |

For peer review only

| | | | |
|---|---|---|---|
| Participants | 13* | (a) Report numbers of individuals at each stage of study—eg numbers potentially eligible, examined for eligibility, confirmed eligible, included in the study, completing follow-up, and analysed | 5,9,10 |
| | | (b) Give reasons for non-participation at each stage | 5, 9, 10 |
| | | (c) Consider use of a flow diagram | |
| Descriptive data | 14* | (a) Give characteristics of study participants (eg demographic, clinical, social) and information on exposures and potential confounders | 9, 21 |
| | | (b) Indicate number of participants with missing data for each variable of interest | 8,9 |
| | | (c) Summarise follow-up time (eg, average and total amount) | 5,6 |
| Outcome data | 15* | Report numbers of outcome events or summary measures over time | 5,6 |
| Main results | 16 | (*a*) Give unadjusted estimates and, if applicable, confounder-adjusted estimates and their precision (eg, 95% confidence interval). Make clear which confounders were adjusted for and why they were included | 9,10, 23 |
| | | (*b*) Report category boundaries when continuous variables were categorized | |
| | | (*c*) If relevant, consider translating estimates of relative risk into absolute risk for a meaningful time period | |
| Other analyses | 17 | Report other analyses done—eg analyses of subgroups and interactions, and sensitivity analyses | 24,27 |
| **Discussion** | | | |
| Key results | 18 | Summarise key results with reference to study objectives | |
| **Limitations** | | | 10,11 |
| Interpretation | 20 | Give a cautious overall interpretation of results considering objectives, limitations, multiplicity of analyses, results from similar studies, and other relevant evidence | 11-13 |
| Generalisability | 21 | Discuss the generalisability (external validity) of the study results | 11-13 |
| **Other information** | | | |
| Funding | 22 | Give the source of funding and the role of the funders for the present study and, if applicable, for the original study on which the present article is based | 14 |

*Give information separately for cases and controls in case-control studies and, if applicable, for exposed and unexposed groups in cohort and cross-sectional studies.

**Note:** An Explanation and Elaboration article discusses each checklist item and gives methodological background and published examples of transparent reporting. The STROBE checklist is best used in conjunction with this article (freely available on the Web sites of PLoS Medicine at http://www.plosmedicine.org/, Annals of Internal Medicine at http://www.annals.org/, and Epidemiology at http://www.epidem.com/). Information on the STROBE Initiative is available at www.strobe-statement.org.

For peer review only

BMJ Open

# Lifetime cigarette smoking and chronic widespread and regional pain in later adulthood: Evidence from the 1946 British birth cohort study

| | |
|---|---|
| Journal: | *BMJ Open* |
| Manuscript ID | bmjopen-2018-021896.R2 |
| Article Type: | Research |
| Date Submitted by the Author: | 18-Jun-2018 |
| Complete List of Authors: | Bendayan, Rebecca; Institute of Psychiatry, Psychology & Neuroscience (IoPPN), King's College London, Dept. Biostatistics and Health Informatics<br>Cooper, Rachel; UCL, MRC Unit for Lifelong Health and Ageing at UCL<br>Muthuri, Stella; MRC Unit for Lifelong Health and Ageing, |
| <b>Primary Subject Heading</b>: | Epidemiology |
| Secondary Subject Heading: | Smoking and tobacco, Public health, Rheumatology |
| Keywords: | Chronic Pain, Cigarette Smoking, Life Course Epidemiology, Birth Cohort |
| | |

SCHOLARONE™
Manuscripts

For peer review only

**Lifetime cigarette smoking and chronic widespread and regional pain in later adulthood: Evidence from the 1946 British birth cohort study**

Rebecca Bendayan[1;2], Rachel Cooper[1], Stella G Muthuri[1]

[1] MRC Unit for Lifelong Health and Ageing at UCL, 33 Bedford Place, London, WC1B 5JU, UK

[2] Dept. Biostatistics and Health Informatics, Institute of Psychiatry, Psychology & Neuroscience (IoPPN), King's College London, De Crespigny Park, London, SE5 8AF, United Kingdom

Correspondence to: Rebecca Bendayan, rebecca.bendayan@kcl.ac.uk

The Corresponding Author has the right to grant on behalf of all authors and does grant on behalf of all authors, a worldwide licence to the Publishers and its licensees in perpetuity, in all forms, formats and media (whether known now or created in the future), to i) publish, reproduce, distribute, display and store the Contribution, ii) translate the Contribution into other languages, create adaptations, reprints, include within collections and create summaries, extracts and/or, abstracts of the Contribution, iii) create any other derivative work(s) based on the Contribution, iv) to exploit all subsidiary rights in the Contribution, v) the inclusion of electronic links from the Contribution to third party material where-ever it may be located; and, vi) licence any third party to do any or all of the above.

Key words: Chronic Pain; Cigarette Smoking; Life Course Epidemiology; Birth Cohort



For peer review only - http://bmjopen.bmj.com/site/about/guidelines.xhtml

**Abstract**

**Objective:** To examine whether different lifetime patterns of cigarette smoking are associated with chronic widespread and regional pain at age 68.

**Design:** Prospective cohort study

**Setting:** England, Scotland and Wales

**Participants:** Up to 2347 men and women from the Medical Research Council National Survey of Health and Development, who have been followed up since birth in 1946 and provided sufficient information on cigarette smoking across adulthood to be classified as never smoker, predominantly non-smoker, predominantly smoker or lifelong smoker; and pain assessment at age 68.

**Primary outcome measures:** Pain was self-reported at age 68, and chronic widespread pain (CWP) was defined according to American College of Rheumatology criteria. Participants who reported having pain for ≥3 months but who did not meet the CWP definition were classified as having chronic regional pain (CRP); those who reported pain which had lasted for < 3 months were classified as 'other' pain. No pain was the reference group.

**Results:** Findings from multinomial logistic regression models indicated that compared with never smokers, predominantly non-smokers, predominantly smokers and lifelong smokers all had an increased risk of CWP; Relative-Risk-Ratios = 1.70 [95%CI 1.16 – 2.49]; 2.10 [95%CI 1.34 – 3.28] and 1.88 [95%CI 0.99 – 3.57], respectively, after adjusting for sex, own occupational class, educational level, body mass index, leisure time physical activity, alcohol intake, longstanding illness and symptoms of anxiety and depression. No association was observed between smoking history and CRP or other pain.

**Conclusions:** These results suggest that exposure to cigarette smoking at any stage in adulthood was associated with higher risk of CWP in later adulthood; highlighting the ongoing importance of smoking prevention programmes. It also suggests that assessment of



For peer review only - http://bmjopen.bmj.com/site/about/guidelines.xhtml

lifetime smoking behaviour may be more useful in identifying those at greater risk of CWP in later life than assessment of current smoking status.

**Strengths and limitations of this study**

This study uses prospective data on smoking behaviour ascertained across adulthood (from age 20 to 68) providing us with the opportunity to characterise different lifetime patterns of smoking.

- This study distinguishes chronic widespread pain from regional pain and examines the risk of both.
- Chronic pain was assessed at a single time point so we were not able to establish temporality.
- Lifelong smokers were under-represented in the study sample as they had greater rates of all-cause mortality.
- Despite losses to follow up over the 69 years of the study, the sample has remained broadly representative of the national population of England, Scotland and Wales born at a similar time.



For peer review only - http://bmjopen.bmj.com/site/about/guidelines.xhtml

## Introduction

Chronic pain is highly prevalent in older populations and has major adverse impacts on quality of life, wellbeing and activity participation[1-4]. Identifying modifiable risk factors associated with pain may inform interventions to prevent and/or alleviate its consequences in later life. Cigarette smoking is one of many modifiable behavioural risk factors that may play a role in the development and progression of chronic pain. While this risk factor has already been widely studied[5,6] with most longitudinal studies reporting weak or null associations[7-10], the majority of these have been in populations younger than 65.

Moreover, even when studies have included older adults these have usually only considered a single assessment of smoking status (either at baseline or at the current or most recent time point). This information has then been used to classify individuals as current, former or never smokers, or as ever or never smokers. This is unlikely to adequately capture the variability in lifetime exposure to smoking and its associated health risks due to changes in smoking behaviour across adulthood. Distinguishing people with different lifetime smoking histories and investigating differences in their health prospects may be especially important in older adults; although the prevalence of smoking has steadily declined across all age groups over the last few decades, the baby boom generation who are currently reaching old age were at high risk of exposure to smoking earlier in adulthood[11]. In addition, they are likely to have been exposed to anti-smoking campaigns and comprehensive tobacco control policies early enough in adulthood to have potentially benefited from the subsequent reductions in risk of smoking related diseases linked to premature mortality[12]. While people with some history of smoking are therefore now more likely to reach old age than they would have been in previous generations, whether they reach old age with more chronic conditions and symptoms such as pain remains to be established.


For peer review only - http://bmjopen.bmj.com/site/about/guidelines.xhtml

Using data from the MRC National Survey of Health and Development (NSHD), which has assessed smoking status at multiple time points across adulthood, this paper aims to examine whether different lifetime patterns of cigarette smoking are associated with chronic widespread and regional pain at age 68.

## Methods

### Study population

The MRC NSHD is a socially stratified population sample of 5362 single, legitimate births that occurred in England, Wales and Scotland in one week of March 1946. Participants have been prospectively followed from birth, and participation rates have remained high[13-16]. At the 24th data collection, conducted between 2014 and 2015 when study members were aged 68-69, study participants were first asked to complete a postal questionnaire at age 68 before then being invited to have a home visit by a research nurse at age 69[15]. Of the 2816 people in the target sample living in England, Scotland and Wales, 2370 (84.2%) completed a postal questionnaire. In addition, a postal questionnaire was sent to 126 study members living abroad who remain in contact with the study of whom 83 (65.9%) returned a questionnaire. No attempt was made to contact the remaining 2420 study members: 957 (17.8%) had already died, 620 (11.6%) had previously withdrawn from the study, 448 (8.3%) had emigrated and were no longer in contact with the study, and 395 (7.4%) had been untraceable for more than five years.

Ethical approval for the data collection at age 68-69 was obtained from the Queen Square Research Ethics Committee (14/LO/1073) and the Scotland A Research Ethics Committee (14/SS/1009) and written informed consent was obtained from study members.



For peer review only - http://bmjopen.bmj.com/site/about/guidelines.xhtml

**Patient and Public involvement statement**

Participants have a lifelong association with NSHD. Over the 72 years of the study, the research team has increasingly involved participants, in line with changing norms about conducting cohort studies, starting at age 16 (in 1962) with the annual dissemination of study findings in birthday cards. Participants receive personal letters whenever they raise queries or provide additional comments, including suggestions for new topics to study. In the last ten years, the research team has increased the level of participant involvement through invitations to study events (65th and 70th birthday celebrations), public engagement activities, and focus groups to discuss clinical sub-studies. When piloting new questionnaires and assessments, we recruit patients from GP practices or from the UCLH Patient Public Involvement (PPI) group, and take into account their feedback when designing the mainstage fieldwork.

**Assessment of pain**

Information on pain was ascertained in the postal questionnaire administered at age 68. Participants were asked whether they had experienced any ache or pain in the last month which had lasted for one day or longer, not including pain occurring during the course of a feverish illness such as flu. Those who reported pain were then asked to report whether or not they had experienced this pain for at least 3 months and to shade the location of their pain using a four-view body manikin. Using American College of Rheumatology criteria[17] chronic widespread pain (CWP) was defined as pain present for 3 months or longer, both above and below the waist; on both the left and right side of the body; and in the axial skeleton. Participants who reported having pain for at least 3 months but who did not meet the CWP definition were classified as having chronic regional pain (CRP) and those who reported pain that had lasted for less than 3 months were classified as having ‘other’ pain[18].



For peer review only - http://bmjopen.bmj.com/site/about/guidelines.xhtml

**Assessment of smoking**

Data on cigarette smoking were obtained on eight occasions across adulthood using postal questionnaires (at ages 20, 25, 31, 60-64, 68-69) and nurse interviews (at ages 36, 43, 53). At each of these ages, study members were asked whether they currently smoked cigarettes. Participants who provided a negative response were asked whether they had ever smoked. This information was used to construct a set of variables indicating smoking status at each age from 20 to 68, coded as current, former and never smoker. Using these 3-category variables, a lifetime smoking history variable was then constructed according to Clennell et al.[19]. Using this approach, we included those who provided smoking data for at least 3 waves and for whom missing data were not sequential at 3 or more waves (n=3705). Based on their reported smoking status at each age across adulthood, participants were grouped into one of four categories to summarise their smoking history: (1) participants who reported being non-smokers at all available data collections were classified as 'never smokers'; (2) participants who reported being smokers at all available data collections were classified as 'lifelong smokers'; participants who were classified as smokers at some ages and as ex or never smokers at other ages were classified as: (3) 'predominantly non-smoker' if they were a non-smoker on at least 3 occasions and more often reported being a non-smoker than a smoker; (4) 'predominantly smoker' if they reported being a smoker on 4 or more occasions and more often reported being a smoker than a non-smoker. For example, if a study member reported being a smoker on five occasions and an ex-smoker on two occasions, they were classified as 'predominantly smoker' whereas if they reported the reverse they were classified as 'predominantly non-smoker'[19].

**Covariates**



For peer review only - http://bmjopen.bmj.com/site/about/guidelines.xhtml

Potential confounders were selected a priori based on previous research on chronic pain and health behaviours[20-23]. These were sex, socioeconomic position (indicated by own occupational class (which also allows us to distinguish between manual and non-manual work) and educational level), body mass index (BMI), other health behaviours (leisure time physical activity and alcohol intake), longstanding illness and symptoms of anxiety and depression. Unless otherwise specified these were assessed at age 68. Own occupation at age 53 years (or if not available, the most recent measure in adulthood) was categorised according to the Registrar General's social classification into three groups: high (I (Professional) or II (Managerial and technical)); middle (IIINM (skilled non-manual [NM]) or IIIM (skilled manual [M])); low (IV (partly skilled manual) or V (unskilled manual)). Highest educational level achieved by age 26 was categorised into three groups: no qualifications; up to O-level or equivalent; A level or equivalent and above. BMI ($kg/m^2$) was calculated from height (m) and weight (kg) measured by a trained nurse during the clinical assessment at age 69. Physical activity was assessed through self-reports of the level of participation in sports, vigorous leisure activities or exercise grouped as: inactive, moderately active (1-4 times/month) or most active (5 times or more/month). Similarly, alcohol intake in the last year was self-reported as never, only on special occasions, monthly or less, 2-4 times per month, 2-3 times per week, or 4 or more times per week. In addition, participants were asked whether they had any longstanding illness or health problems which had lasted, or were expected to last for 6 months or more. Symptoms of anxiety and depression were assessed using the 28-item General Health Questionnaire (GHQ-28). Each GHQ-28 item was coded using the General Scoring Method (i.e. 0 for response choices 1 and 2; and as 1 or response choices 3 and 4). Symptoms of anxiety and depression were indicated by summing responses to all items and applying a threshold for caseness of 5 or more[24].



For peer review only - http://bmjopen.bmj.com/site/about/guidelines.xhtml

**Statistical analyses**

Multinomial logistic regression models were used to examine the associations between lifetime smoking history and pain outcomes (with no pain as the reference category). Models were first adjusted for sex and formal tests of interaction between sex and lifetime smoking history were undertaken. As no evidence of interactions with sex were found, subsequent models were sex adjusted. We then adjusted for each set of covariates in turn before adjusting for all covariates simultaneously in a final model. All models included the maximum number of participants with complete data on lifetime smoking history and pain (n=2347). To reduce data loss, covariates with missing values (i.e. educational attainment [n=114], occupational class [n=6], BMI [n=435], leisure time physical activity [n=25], alcohol intake [n=25], longstanding illness or health problems [n=24] and GHQ-28 [n=422] were imputed using multiple imputation by chained equations[25]. Analyses were performed across 20 imputed datasets and combined using Rubin's rules. In sensitivity analyses, models were rerun on the sample with complete data on all covariates (n=1759). All analyses were conducted using STATA v14.1.

**Results**

Of the total analytical sample of 2347, 1304 (55.6%) reported pain, with 10.6% reporting CWP, 30.3% reporting CRP, and 14.7% reporting 'other pain'. More women than men reported CWP (13.2% for women vs 7.7% for men, p<.001). Overall, the prevalence of smoking declined across adulthood from 55.8% for men and 46.8% of women at age 20 to 9.9% for men and 7.8% for women by age 68 (p-values for sex differences at both ages<.001) (Supplementary Table 1). The prevalence of lifelong non-smokers was 34.3% for women and 26.3% for men. Of those classified as predominantly non-smokers, 61.6% had stopped



For peer review only - http://bmjopen.bmj.com/site/about/guidelines.xhtml

smoking at least once by age 25 and only 0.5% were smokers at the most recent report. Of those classified as predominantly smokers, 88.9% were smokers at age 25, but only 14.2% were still smokers at the most recent report. Descriptive statistics are shown in Table 1. All covariates were associated with pain outcomes in the expected directions.

PLEASE, INSERT TABLE 1 AROUND HERE

In sex-adjusted models, predominantly non-smokers, predominantly smokers, and lifelong smokers all had a higher likelihood of CWP, compared with lifelong non-smokers (Relative-Risk Ratios (RRR) of CWP vs no pain=1.80 (95%CI 1.26 – 2.58), 2.64 (95%CI 1.74 – 3.99) and 2.27 (95%CI 1.25 – 4.13), respectively (Table 2, Model 1). Adjustment for each set of covariates had only limited impact and associations were maintained in fully-adjusted models; RRRs = 1.70 (95%CI 1.16 – 2.49) for predominantly non-smokers, 2.10 (95%CI 1.34 – 3.28) for predominantly smokers and 1.88 (95%CI 0.99 – 3.57) for lifelong smokers (Table 2, Model 6). No associations were observed between lifetime smoking history and CRP or other pain (Table 2).

PLEASE, INSERT TABLE 1 AROUND HERE

When analyses were restricted to those with complete data (n=1759), we found similar results and the overall conclusions remained the same (Supplemental Table 2).

**Discussion**

The main aim of this study was to examine whether different patterns of cigarette smoking over the life course were associated with CWP and CRP at age 68 in a nationally representative birth cohort. Our results showed that exposure to cigarette smoking at any time point across adulthood was associated with increased risk of CWP at age 68, even after adjustment for a range of potential confounders and mediators. However, there was no evidence of associations between lifetime cigarette smoking and CRP or other pain.



For peer review only - http://bmjopen.bmj.com/site/about/guidelines.xhtml

*Comparisons with other studies*

Our finding of an association between different patterns of lifetime smoking and CWP in older adults builds on findings from other previous studies of chronic pain in older adults. For example, in a longitudinal study of British adults aged 50 years or older, individuals who were current smokers had higher odds of pain at baseline but there was no evidence for increased likelihood of new-onset widespread pain over 3 years of follow-up when former and current smokers were combined in one group and compared with never smokers[23]. Likewise, a cross-sectional study found that smoking status was only associated with chronic pain prevalence when current and former smokers were combined in one group and compared with never smokers among people aged 65 years and older in Sweden[26]. By using data collected prospectively across adulthood we were able to distinguish different lifetime patterns of cigarette smoking and go beyond previous research. This has strengths when compared with other studies that have relied on single assessments of smoking and may therefore not adequately or reliably capture all relevant lifetime exposure.

*Potential explanations of findings*

Our finding of increased risk of CWP among all smoking groups (i.e. predominantly non-smokers, predominantly smokers and lifelong smokers), when compared with never smokers suggests that cigarette smoking even for a short period of time over the life course may have a long-lasting impact on the probability of reporting CWP in later adulthood. One plausible explanation for this finding is that smoking may produce changes in central nervous system function that increase susceptibility to pain the effects of which continue to persist after smoking cessation[27]. Another consideration is that the associations between smoking and pain may be bi-directional; not only is there evidence that tobacco smoking may be a risk



For peer review only - http://bmjopen.bmj.com/site/about/guidelines.xhtml

factor for the development and exacerbation of pain but also that pain may serve to motivate smoking[7]. For this reason, it is possible that some individuals in our study may have developed CWP in early adulthood and used smoking as a coping strategy for this with this then causing them to experience greater difficulty quitting[28].

That associations were observed between lifetime smoking and CWP but not with CRP or other pain suggests that any influence of smoking on pain is likely to be operating via mechanisms related specifically to the development of CWP. This is consistent with the idea that CWP and CRP may have different underlying aetiologies[7]. For example, some studies have suggested that nicotine may alter pain regulatory mechanisms (e.g. potential alteration of pain processing or cause structural changes to other systems) which might have long-term consequences for painful chronic conditions[29]. The associations between lifetime smoking and CWP were only partially attenuated after adjustment for a range of factors including sex, socioeconomic position, body size, other health behaviour, longstanding illness and symptoms of anxiety and depression. This suggests that there might be other relevant explanatory variables. For example, although affective symptoms have been taken into consideration in our study some psychosocial models have highlighted the role of pain-related stress, fear or anxiety sensitivity in the transition from acute to chronic pain[7,30,31]. These pain specific measures were not available in our data and therefore future studies are encouraged to include these and examine their association with CWP onset and smoking.

*Methodological considerations*

One of the main strengths of our study was the use of data from a large nationally representative sample of older adults with prospective ascertainment of smoking status across adulthood which allowed us to characterise different lifetime patterns of smoking. Although we were unable to validate self-reports of smoking using cotinine measurements or reliably distinguish between light and heavy smokers, our approach does still have important


For peer review only - http://bmjopen.bmj.com/site/about/guidelines.xhtml

strengths when compared with the commonly used categorisation of never, ex or current smoker based on self-reported smoking at a single time point. While the use of recalled information on quitting attempts between assessments could have introduced bias, when we performed sensitivity analyses to check for potential inconsistencies in reporting and compared results with retrospective measures on time since quitting for former smokers there were no differences in findings. However, the observed increase in prevalence of never smokers at older ages (Supplementary table 2) suggests that smokers were more likely than non-smokers to be lost to follow-up. This is partly explained by the fact that lifelong smokers had much greater rates of all-cause mortality across adulthood up to age 69 (Supplementary table 3); survival bias or differences in statistical power may therefore explain why predominantly smokers were found to have higher relative risks of CWP than lifelong smokers when both groups were compared with never smokers. However, another potential explanation of this finding which needs to be considered is the possibility that lifelong smokers may be less likely to experience CWP than predominantly smokers due to psychological or physiological effects.

Another potential limitation of our analyses is that we cannot rule out reverse causation as chronic pain was only assessed at one time point. As noted above, recent research has highlighted that the association between smoking and pain is likely to be bi-directional[28,32] and so future longitudinal studies with prospective ascertainment of both smoking and pain across life should be performed. Such studies would also benefit from assessment of pain severity and information on potential causes of pain, such as history of accident, which we were not able to take account of in our analyses but which may provide important additional insights. Although our findings are generalizable to the current generation of adults reaching old age in the UK, changing trends in smoking behaviour over the last 70 years[11,33], should be



For peer review only - http://bmjopen.bmj.com/site/about/guidelines.xhtml

considered and further research is needed to examine whether similar associations will be observed in more recently born cohorts who will have experienced different selection effects into smoking and different lifetime patterns of exposure to smoking.

*Conclusions*

Our results highlight the role of smoking at any time point across adulthood as an independent risk factor for CWP in later adulthood, after considering socio-economic, psychosocial and health-related factors. The evidence provided for the long-lasting effect of smoking on this condition highlights the potential benefit of considering smoking history rather than current smoking status when identifying those at greater risk of CWP in later life especially as opportunities to prevent CWP may be missed if only current smokers are classified as higher risk. These findings are relevant from a clinical and public health perspective, as smoking is a modifiable risk factor and our findings suggest that its primary prevention may be key.

For peer review only



For peer review only - http://bmjopen.bmj.com/site/about/guidelines.xhtml

**Acknowledgements:** The authors are grateful to NSHD study members for their continuing support. We also thank members of the NSHD scientific and data collection teams who have been involved in NSHD data collections.

**Funding:** This work was supported by the UK Medical Research Council who provide support for RC and SM (Programme code MC_UU_12019/4) and fund the MRC National Survey of Health and Development. RB was supported in part by grant MR/R016372/1 for the King's College London MRC Skills Development Fellowship programme funded by the UK Medical Research Council (MRC) and by grant IS-BRC-1215-20018 for the National Institute for Health Research (NIHR) Biomedical Research Centre at South London and Maudsley NHS Foundation Trust and King's College London. The views expressed are those of the authors and not necessarily those of the MRC, NHS, the NIHR or the Department of Health and Social Care. The funders of the study had no role in the study design, data collection, data analysis, data interpretation, writing of the report or the decision to submit the article for publication.

**Open access:** This is an Open Access article distributed in accordance with the terms of the Creative Commons Attribution (CC BY 3.0) license, which permits others to distribute, remix, adapt and build upon this work, for commercial use, provided the original work is properly cited. See: http://creativecommons.org/licenses/by/3.0.

**Declaration of competing interests:** All authors have completed the ICMJE uniform disclosure form at www.icmje.org/coi_disclosure.pdf and declare: RC and SM received financial support from the UK Medical Research Council for the submitted work; no financial relationships with any organisations that might have an interest in the submitted work in the previous three years; no other relationships or activities that could appear to have influenced the submitted work.

For peer review only



For peer review only - http://bmjopen.bmj.com/site/about/guidelines.xhtml

**Author contributions:** RB and SGM conceived the idea for this study and along with RC developed the study objectives. RC was part of the team responsible for the 68-69 year data collection. RB and SGM undertook statistical analyses. All authors contributed to the interpretation of the data. RB and SGM drafted the article and all authors contributed to its critical revision and provided final approval of the version to be published. RB and SGM are the guarantors and accept full responsibility for the work and the conduct of the study, had access to the data, and controlled the decision to publish. All authors had full access to all of the data (including statistical reports and tables) in the study and can take responsibility for the integrity of the data and the accuracy of the data analysis.

**Transparency declaration:** RB and SGM affirm that the manuscript is an honest, accurate, and transparent account of the study being reported and that no important aspects of the study have been omitted.

**Data sharing statement:** Data are available on request to the NSHD Data Sharing Committee. NSHD data sharing policies and processes meet the requirements and expectations of MRC policy on sharing of data from population and patient cohorts: http://www.mrc.ac.uk/research/research-policy-ethics/data-sharing/policy/. Data requests should be submitted to mrclha.swiftinfo@ucl.ac.uk ; further details can be found at http://www.nshd.mrc.ac.uk/data.aspx DOI: 10.5522/NSHD/Q101;10.5522/NSHD/Q102; 10.5522/NSHD/Q103.

These policies and processes are in place to ensure that the use of data from this 71 year old national birth cohort study is within the bounds of consent given previously by study members, complies with MRC guidance on ethics and research governance, and meets rigorous MRC data security standards.



For peer review only - http://bmjopen.bmj.com/site/about/guidelines.xhtml

**References:**


1. Gardener EA, Huppert FA, Guralnik JM, Melzer D. Middle□Aged and Mobility□ Limited: Prevalence of Disability and Symptom Attributions in a National Survey. *J Gen Intern Med* 2006;*21*(10):1091-1096. doi:10.1111/j.1525-1497.2006.00564.x
2. Leveille SG, Bean J, Ngo L, *et al.* (2007). The pathway from musculoskeletal pain to mobility difficulty in older disabled women. *Pain* 2007;*128*(1): 69-77. doi:10.1016/j.pain.2006.08.031
3. Leveille SG, Fried L, Guralnik JM. Disabling symptoms. *J Gen Intern Med* 2002;*17*(10):766-773. doi:10.1046/j.1525-1497.2002.20229.x
4. Patel KV, Guralnik JM, Dansie EJ, Turk DC. Prevalence and impact of pain among older adults in the United States: findings from the 2011 National Health and Aging Trends Study. *Pain* 2013;*154*(12):2649-2657. doi:10.1016/j.pain.2013.07.029
5. Ditre JW, Brandon TH, Zale EL, Meagher MM. Pain, nicotine, and smoking: research findings and mechanistic considerations. *Psychol Bull* 2011;*137*(6):1065. doi:10.1037/a0025544
6. Parkerson HA, Zvolensky MJ, Asmundson GJ. Understanding the relationship between smoking and pain. *Expert Rev Neurother* 2013;*13*(12):1407-1414. doi:10.1586/14737175.2013.859524
7. Shiri R, Karppinen J, Leino-Arjas P, *et al.* The Association between Smoking and Low Back Pain: A Meta-analysis. *Am J Med* 2010;*123*(1):87.e7-87.e35. doi:10.1016/j.amjmed.2009.05.028
8. Mundal I, Gråwe R, Bjørngaard J, *et al.* Prevalence and long-term predictors of persistent chronic widespread pain in the general population in an 11-year prospective study: the HUNT study. *BMC Musculoskelet Disord* 2014, 15(1), 213. doi:10.1186/1471-2474-15-213




For peer review only - http://bmjopen.bmj.com/site/about/guidelines.xhtml

9. Volkman J, DeRicke EC, Driscoll MA, *et al.* Smoking status and pain intensity among OEF/OIF/OND veterans. *Pain Medicine* 2015;16(9):1690-1696. doi:10.1111/pme.12753

10. John U, Hanke M, Meyer, C, Volzke, H, Baumeister S, Alte D. Tobacco smoking in relation to pain in a national general population survey. *Prev Med* 2006;43(6): 477-481. doi: 10.1016/j.ypmed.2006.07.005

11. Davy M. Time and generational trends in smoking among men and women in Great Britain, 1972–2004/05. *Health Statistics Quarterly* 2006;*32*:35-43.

12. Pirie K, Peto R, Reeves GK*, et al.* The 21st century hazards of smoking and benefits of stopping: a prospective study of one million women in the UK. *Lancet* 2013;*381*(9861):133-141. doi:10.1016/S0140-6736(12)61720-6

13. Wadsworth M, Kuh D, Richards M, Hardy R. Cohort Profile: The 1946 National Birth Cohort (MRC National Survey of Health and Development). *Int J Epidemiol* 2006;35(1):49-54. doi:10.1093/ije/dyi201

14. Kuh D, Pierce M, Adams J, *et al.* Cohort profile: updating the cohort profile for the MRC National Survey of Health and Development: a new clinic-based data collection for ageing research. *Int J Epidemiol* 2011;40(1):e1-9. doi:10.1093/ije/dyq231

15. Kuh D, Wong A, Shah I, *et al*. The MRC National Survey of Health and Development reaches age 70: maintaining participation at older ages in a birth cohort study. *Eur J Epidemiol* 2016;31(11):1135-1147. doi:10.1007/s10654-016-0217-8

16. Stafford M, Black S, Shah I, *et al.* Using a birth cohort to study ageing: representativeness and response rates in the National Survey of Health and Development. *Eur J Ageing* 2013;*10*(2):145-157. doi:10.1007/s10433-013-0258-8

17. Wolfe F, Smythe HA, Yunus MB, *et al.* The American College of Rheumatology 1990 criteria for the classification of fibromyalgia. *Arthritis & Rheumatology* 1990;*33*(2): 160-172. doi:10.1002/art.1780330203



For peer review only - http://bmjopen.bmj.com/site/about/guidelines.xhtml

18. Muthuri SG, Kuh D, Bendayan R, *et al*. Chronic physical illness in early life and risk of chronic widespread and regional pain at age 68: evidence from the 1946 British birth cohort. *Pain* 2016;*157*(10):2382. doi:10.1097/j.pain.0000000000000663

19. Clennell S, Kuh D, Guralnik J, *et al*. Characterisation of smoking behaviour across the life course and its impact on decline in lung function and all-cause mortality: evidence from a British birth cohort. *J Epidemiol Community Health* 2008;*62*(12):1051-1056. doi:10.1136/jech.2007.068312

20. Bergman S. Psychosocial aspects of chronic widespread pain and fibromyalgia. *Disability and Rehabilitation* 2005;*27*(12):675-683. doi:10.1080/09638280400009030

21. Ferreira PH, Beckenkamp P, Maher CG, *et al.* (2013). Nature or nurture in low back pain? Results of a systematic review of studies based on twin samples. *Eur J Pain* 2013;*17*(7):957-971. doi:10.1002/j.1532-2149.2012.00277.x

22. Junqueira DRG, Ferreira ML, Refshauge K, *et al.* Heritability and lifestyle factors in chronic low back pain: results of the Australian Twin Low Back Pain Study (The AUTBACK study). *Eur J Pain* 2014;*18*(10):1410-1418. doi:10.1002/ejp.506

23. McBeth J, Lacey RJ, Wilkie R. Predictors of New□Onset Widespread Pain in Older Adults: Results From a Population□Based Prospective Cohort Study in the UK. *Arthritis & Rheumatology* 2014;*66*(3):757-767. doi:10.1002/art.38284

24. Goldberg DP, Hillier VF. A scaled version of the General Health Questionnaire. *Psychol Med* 1979;9(1):139-145. doi:10.1017/S0033291700021644

25. Sterne JAC, White IR, Carlin JB, *et al.* Multiple imputation for missing data in epidemiological and clinical research: potential and pitfalls. *BMJ* 2009;338.

26. Jakobsson U, Larsson C. Smoking and chronic pain among people aged 65 years and older. *Pain Practice* 2014;*14*(3):237-244. doi:10.1111/papr.12067



For peer review only - http://bmjopen.bmj.com/site/about/guidelines.xhtml

27. Perkins KA, Grobe JE, Stiller RL, *et al.* Effects of nicotine on thermal pain detection in humans. *Exp Clin Psychopharmacol*;2:95-106.

28. Ditre JW, Kosiba JD, Zale EL, *et al.* Chronic pain status, nicotine withdrawal, and expectancies for smoking cessation among lighter smokers. *Ann Behav Med* 2016;*50*(3):427-435. doi:10.1007/s12160-016-9769-9

29. Shi Y, Weingarten TN, Mantilla CB, *et al.* Smoking and Pain Pathophysiology and Clinical Implications. *The Journal of the American Society of Anesthesiologists* 2010;*113*(4): 977-992.

30. Asmundson GJ, Norton PJ, Vlaeyen JW. Fear-avoidance models of chronic pain: an overview. *Understanding and Treating Fear of Pain* 2004;3-24.

31. Vlaeyen JW, Linton SJ. Fear-avoidance and its consequences in chronic musculoskeletal pain: a state of the art. *Pain* 2000;*85*(3):317-332. doi:10.1016/S0304-3959(99)00242-0

32. Zale EL, Maisto SA, Ditre JW. Anxiety and depression in bidirectional relations between pain and smoking: Implications for smoking cessation. *Behavior Modification* 2016;*40*(1-2):7-28. doi:10.1177/0145445515610744

33. Thun MJ, Carter BD, Feskanich D, et al. 50-year trends in smoking-related mortality in the United States. *N Engl J Med* 2013;*368*(4):351-364. doi:10.1056/NEJMsa1211127

For peer review only



For peer review only - http://bmjopen.bmj.com/site/about/guidelines.xhtml

Table 1: Characteristics of participants from the MRC National Survey of Health and Development by sex at age 68 [Maximum N=2347]

| | Men, N† (%) | Women, N† (%) | P value* |
|---|---|---|---|
| Total | 1,123 (47.9) | 1,224 ( 52.1) | |
| **Pain at age 68** | | | |
| No pain | 537 (47.8) | 506 (41.3) | <.001 |
| Other pain | 184 (16.4) | 162 (13.2) | |
| Chronic Regional Pain | 316 (28.1) | 394 (32.2) | |
| Chronic Widespread Pain | 86 (7.7) | 162 (13.2) | |
| **Lifetime smoking history** | | | |
| Never smoker | 295 (26.3) | 420 (34.3) | <.001 |
| Predominantly non-smoker | 527 (46.9) | 516 (42.2) | |
| Predominantly smoker | 228 (20.3) | 224 (18.3) | |
| Lifelong smoker | 73 (6.5) | 64 (5.2) | |
| **BMI (kg/m$^2$) at age 69** (n=1919) | | | |
| Mean (SD) | 28.0 (4.46) | 28.1 (5.56) | .92 |
| **Occupational class at age 53** | | | |
| High (I/II) | 631 (56.3) | 458 (37.5) | <.001 |
| Middle (IIINM/ IIIM) | 385 (34.4) | 543 (44.5) | |
| Low (IV/V) | 104 (9.3) | 220 (18.0) | |
| **Educational attainment by age 26** | | | |
| A level or equivalent & above | 505 (47.2) | 374 (32.2) | <.001 |
| Up to O level or equivalent | 212 (19.8) | 419 (36.1) | |
| None | 354 (33.1) | 369 (31.8) | |
| **Alcohol intake at age 68** | | | |
| Never | 205 (18.5) | 396 (32.7) | <.001 |
| Monthly or less | 55 (5.0) | 107 (8.8) | |
| 2-4 times per month | 157 (14.1) | 203 (16.8) | |
| 2-3 times per week | 295 (26.6) | 262 (21.6) | |
| ≥4 times per week | 398 (35.9) | 244 (20.1) | |
| **Participation in leisure time physical activity at age 68** | | | |
| Most active( ≥5 times/ month) | 319 (28.7) | 320 (26.4) | .18 |
| Moderately active ( 1-4 times/ month) | 123 (11.1) | 161 (13.3) | |
| Inactive | 669 (60.2) | 730 (60.3) | |
| **Longstanding illness or health problem at age 68** | | | |



For peer review only - http://bmjopen.bmj.com/site/about/guidelines.xhtml

| | Men, N[†] (%) | Women, N[†] (%) | P value[*] |
|---|---|---|---|
| No | 449 (40.3) | 508(42.0) | .38 |
| Yes | 666 (59.7) | 700 (57.9) | |
| **Symptoms of anxiety and depression (GHQ-28) at age 68** | | | |
| No (≤4) | 840 (90.7) | 829 (83.0) | <.001 |
| Yes (>4) | 86 (9.3) | 170 (17.0) | |

*comparison of sexes using student t-test or chi-square tests as appropriate

† Sample restricted to those participants with data on smoking history and pain. N varies due to missing data on covariates

For peer review only



For peer review only - http://bmjopen.bmj.com/site/about/guidelines.xhtml

Table 2. Associations between lifetime smoking history and pain at age 68 (n=2347)

| | CWP vs no pain | | CRP vs no pain | | Other pain vs no pain | |
|---|---|---|---|---|---|---|
| | RRR (95% CI) | P | RRR (95% CI) | P | RRR (95% CI) | P |
| **Model 1** | | | | | | |
| Never smoker | 1 | .0001 | 1 | .25 | 1 | .25 |
| Predominantly non-smoker | 1.80 (1.26 , 2.58) | | 1.08 (0.86 , 1.35) | | 1.14 (0.85 , 1.52) | |
| Predominantly smoker | 2.64 (1.74 , 3.99) | | 1.32 (0.99 , 1.74) | | 1.44 (1.01 , 2.04) | |
| Lifelong smoker | 2.27 (1.25 , 4.13) | | 0.97 (0.62 , 1.51) | | 1.05 (0.60 , 1.84) | |
| **Model 2** | | | | | | |
| Never smoker | 1 | <.001 | 1 | .33 | 1 | .09 |
| Predominantly non-smoker | 1.80 (1.26 , 2.59) | | 1.08 (0.86 , 1.35) | | 1.16 (0.87 , 1.55) | |
| Predominantly smoker | 2.52 (1.65 , 3.84) | | 1.29 (0.97 , 1.71) | | 1.51 (1.06 , 2.17) | |
| Lifelong smoker | 2.11 (1.15 , 3.86) | | 0.94 (0.60 , 1.47) | | 1.13 (0.64 , 1.99) | |
| **Model 3** | | | | | | |
| Never smoker | 1 | <.001 | 1 | .46 | 1 | .17 |
| Predominantly non-smoker | 1.85 (1.28 , 2.66) | | 1.06 (0.84 , 1.33) | | 1.09 (0.82 , 1.47) | |
| Predominantly smoker | 2.43 (1.59 , 3.71) | | 1.22 (0.92 , 1.62) | | 1.37 (0.96 , 1.96) | |
| Lifelong smoker | 2.12 (1.15 , 3.91) | | 0.95 (0.60 , 1.48) | | 1.12 (0.63 , 1.99) | |
| **Model 4** | | | | | | |
| Never smoker | 1 | <.001 | 1 | .45 | 1 | .20 |
| Predominantly non-smoker | 1.69 (1.17 , 2.45) | | 1.05 (0.83 , 1.31) | | 1.12 (0.84 , 1.50) | |
| Predominantly smoker | 2.33 (1.52 , 3.58) | | 1.23 (0.93 , 1.64) | | 1.40 (0.98 , 2.00) | |
| Lifelong smoker | 2.08 (1.12 , 3.86) | | 0.93 (0.59 , 1.46) | | 1.03 (0.59 , 1.81) | |
| **Model 5** | | | | | | |
| Never smoker | 1 | <.001 | 1 | .37 | 1 | .20 |
| Predominantly non-smoker | 1.77 (1.23 , 2.55) | | 1.07 (0.86 , 1.34) | | 1.13 (0.85 , 1.51) | |
| Predominantly smoker | 2.47 (1.62 , 3.76) | | 1.27 (0.96 , 1.68) | | 1.40 (0.99 , 2.00) | |
| Lifelong smoker | 2.13 (1.16 , 3.91) | | 0.93 (0.60 , 1.46) | | 1.02 (0.58 , 1.80) | |
| **Model 6** | | | | | | |
| Never smoker | 1 | .002 | 1 | .90 | 1 | .15 |
| Predominantly non-smoker | 1.70 (1.16 , 2.49) | | 1.01 (0.80 , 1.28) | | 1.10 (0.82 , 1.48) | |
| Predominantly smoker | 2.10 (1.34 , 3.28) | | 1.12 (0.83 , 1.51) | | 1.39 (0.97 , 2.01) | |
| Lifelong smoker | 1.88 (0.99 , 3.57) | | 0.87 (0.54 , 1.38) | | 1.15 (0.64 , 2.06) | |

Note: RRR = relative risk ratio; CWP = chronic widespread pain; CRP = chronic regional pain

Model adjustments:

1: sex

2: sex and SEP (education at age 26, own occupational class)

3: sex and other health behaviours (BMI, alcohol intake and physical activity)

4: sex and longstanding illness or health problems

5: sex and symptoms of anxiety and depression (GHQ-28)

6: all covariates included in models 1 to 5



For peer review only - http://bmjopen.bmj.com/site/about/guidelines.xhtml

Supplementary information

Supplementary Table 1: Summary of smoking history data (i.e. those with at least 1 wave of smoking data and pain at age 68, n=2409)

| | Men, N (%) | Women, N (%) | P value |
|---|---|---|---|
| **Ever smoker, N=2409** | | | |
| No | 310 (26.8) | 431 (34.5) | <.001 |
| Yes | 848 (73.2) | 820 (65.6) | |
| **Smoking history at** | | | |
| **Age 20, n=2074** | | | |
| Current smoker | 551 (55.8) | 508 (46.8) | <.001 |
| Ex-smoker | 129 (13.1) | 145 (13.4) | |
| Never | 308 (31.2) | 433 (39.9) | |
| **Age 25, N=1956** | | | |
| Current smoker | 390 (42.4) | 344 (33.2) | <.001 |
| Ex-smoker | 255 (27.8) | 302 (29.1) | |
| Never | 274 (29.8) | 391 (37.7) | |
| **Age 31, N=2032** | | | |
| Current smoker | 375 (38.5) | 373 (35.3) | .001 |
| Ex-smoker | 336 (34.5) | 318 (30.1) | |
| Never | 263 (27) | 367 (34.7) | |
| **Age 36, N=2129** | | | |
| Current smoker | 301 (29.7) | 298 (26.8) | .001 |



For peer review only - http://bmjopen.bmj.com/site/about/guidelines.xhtml

| | Men, N (%) | Women, N (%) | P value |
|---|---|---|---|
| Ex-smoker | 431 (42.5) | 423 (38) | |
| Never | 283 (27.9) | 393 (35.3) | |
| **Age 43, N=2196** | | | |
| Current smoker | 278 (26.6) | 270 (23.5) | <.001 |
| Ex-smoker | 397 (38) | 312 (27.1) | |
| Never | 371 (35.5) | 568 (49.4) | |
| **Age 53, N=2157** | | | |
| Current smoker | 201 (19.9) | 210 (18.3) | <.001 |
| Ex-smoker | 437 (43.2) | 348 (30.4) | |
| Never | 374 (37.0) | 587 (51.3) | |
| **Age 60-64, N=2027** | | | |
| Current smoker | 109 (11.4) | 120 (11.2) | <.001 |
| Ex-smoker | 452 (47.3) | 367 (34.3) | |
| Never | 395 (41.3) | 584 (54.5) | |
| **Age 68, N=2366** | | | |
| Current smoker | 113 (9.9) | 96 (7.8) | <.001 |
| Ex-smoker | 537 (47.1) | 413 (33.7) | |
| Never | 489 (48.9) | 718 (58.5) | |



For peer review only - http://bmjopen.bmj.com/site/about/guidelines.xhtml

Supplementary Table 2: Associations between lifetime smoking history (vs never smoker) and pain outcomes, n=1759 (complete case analysis)

| | **CWP vs no pain** | | **CRP vs no pain** | | **Other pain vs no pain** | |
|---|---|---|---|---|---|---|
| Model | RRR (95% CI) | P value | RRR (95% CI) | P value | RRR (95% CI) | P value |
| *Sex-adjusted* | | | | | | |
| Never smoker | 1 | .0028 | 1 | .17 | 1 | .18 |
| Predominantly non-smoker | 1.83 (1.22, 2.75) | | 1.14 (0.89 , 1.48) | | 1.11 (0.79, 1.57) | |
| Predominantly smoker | 2.39 (1.49 , 3.84) | | 1.34 (0.97 , 1.84) | | 1.53 (1.02 , 2.29) | |
| Lifelong smoker | 1.60 (0.75 , 3.46) | | 0.78 (0.45 , 1.37) | | 0.91 (0.45, 1.85) | |
| *Fully-adjusted** | | | | | | |
| Never smoker | 1 | .024 | 1 | .35 | 1 | .23 |
| Predominantly non-smoker | 1.76 (1.15 , 2.70) | | 1.10 (0.84, 1.44) | | 1.09 (0.77, 1.55) | |
| Predominantly smoker | 2.09 (1.26 , 3.47) | | 1.20 (0.86, 1.69) | | 1.52 (0.99, 2.31) | |
| Lifelong smoker | 1.47 (0.65 , 3.31) | | 0.73 (0.41 , 1.31) | | 0.99 (0.48 , 2.06) | |

*Adjusted for all covariates (sex, own occupational class, educational level, BMI, leisure time physical activity, alcohol intake, longstanding illness and symptoms of anxiety and depression.)



For peer review only - http://bmjopen.bmj.com/site/about/guidelines.xhtml

Supplementary Table 3: Hazard ratio for all-cause mortality by lifetime smoking history

| | Sex adjusted | | Adjusted* | |
|---|---|---|---|---|
| | HR (95% CI) | P value | HR (95% CI) | P value |
| Never smoker | 1 | | 1 | |
| Predominantly non-smoker | 1.21 (0.89 1.64) | .22 | 1.22 (0.90 – 1.65) | .21 |
| Predominantly smoker | 1.59 (1.14 – 2.22) | .007 | 1.52 (1.09 – 2.13) | .014 |
| Lifelong smoker | 5.12 (3.79 – 6.90) | <.001 | 4.71 (3.48 – 6.39) | <.001 |

*adjusted for sex, father's occupational class and own occupational class

For peer review only

## STROBE 2007 (v4) Statement—Checklist of items that should be included in reports of *cohort studies*

| Section/Topic | Item # | Recommendation | Reported on page # |
|---|---|---|---|
| **Title and abstract** | 1 | (*a*) Indicate the study's design with a commonly used term in the title or the abstract | 1, 2 |
| | | (*b*) Provide in the abstract an informative and balanced summary of what was done and what was found | 2, 3 |
| **Introduction** | | | |
| Background/rationale | 2 | Explain the scientific background and rationale for the investigation being reported | 4 |
| Objectives | 3 | State specific objectives, including any prespecified hypotheses | 5 |
| **Methods** | | | |
| Study design | 4 | Present key elements of study design early in the paper | 5 |
| Setting | 5 | Describe the setting, locations, and relevant dates, including periods of recruitment, exposure, follow-up, and data collection | 5,6 |
| Participants | 6 | (*a*) Give the eligibility criteria, and the sources and methods of selection of participants. Describe methods of follow-up | 5 |
| | | (*b*) For matched studies, give matching criteria and number of exposed and unexposed | |
| Variables | 7 | Clearly define all outcomes, exposures, predictors, potential confounders, and effect modifiers. Give diagnostic criteria, if applicable | 6-8 |
| Data sources/ measurement | 8* | For each variable of interest, give sources of data and details of methods of assessment (measurement). Describe comparability of assessment methods if there is more than one group | 8 |
| Bias | 9 | Describe any efforts to address potential sources of bias | 8,9 |
| Study size | 10 | Explain how the study size was arrived at | 9 |
| Quantitative variables | 11 | Explain how quantitative variables were handled in the analyses. If applicable, describe which groupings were chosen and why | 9, 10 |
| Statistical methods | 12 | (*a*) Describe all statistical methods, including those used to control for confounding | 9,10 |
| | | (*b*) Describe any methods used to examine subgroups and interactions | 9,10 |
| | | (*c*) Explain how missing data were addressed | 9,10 |
| | | (*d*) If applicable, explain how loss to follow-up was addressed | 10 |
| | | (*e*) Describe any sensitivity analyses | 10 |
| **Results** | | | |

For peer review only

| | | | |
|---|---|---|---|
| Participants | 13* | (a) Report numbers of individuals at each stage of study—eg numbers potentially eligible, examined for eligibility, confirmed eligible, included in the study, completing follow-up, and analysed | 5,9,10 |
| | | (b) Give reasons for non-participation at each stage | 5, 9, 10 |
| | | (c) Consider use of a flow diagram | |
| Descriptive data | 14* | (a) Give characteristics of study participants (eg demographic, clinical, social) and information on exposures and potential confounders | 9, 21 |
| | | (b) Indicate number of participants with missing data for each variable of interest | 8,9 |
| | | (c) Summarise follow-up time (eg, average and total amount) | 5,6 |
| Outcome data | 15* | Report numbers of outcome events or summary measures over time | 5,6 |
| Main results | 16 | (*a*) Give unadjusted estimates and, if applicable, confounder-adjusted estimates and their precision (eg, 95% confidence interval). Make clear which confounders were adjusted for and why they were included | 9,10, 23 |
| | | (*b*) Report category boundaries when continuous variables were categorized | |
| | | (*c*) If relevant, consider translating estimates of relative risk into absolute risk for a meaningful time period | |
| Other analyses | 17 | Report other analyses done—eg analyses of subgroups and interactions, and sensitivity analyses | 24,27 |
| **Discussion** | | | |
| Key results | 18 | Summarise key results with reference to study objectives | |
| **Limitations** | | | 10,11 |
| Interpretation | 20 | Give a cautious overall interpretation of results considering objectives, limitations, multiplicity of analyses, results from similar studies, and other relevant evidence | 11-13 |
| Generalisability | 21 | Discuss the generalisability (external validity) of the study results | 11-13 |
| **Other information** | | | |
| Funding | 22 | Give the source of funding and the role of the funders for the present study and, if applicable, for the original study on which the present article is based | 14 |

*Give information separately for cases and controls in case-control studies and, if applicable, for exposed and unexposed groups in cohort and cross-sectional studies.

**Note:** An Explanation and Elaboration article discusses each checklist item and gives methodological background and published examples of transparent reporting. The STROBE checklist is best used in conjunction with this article (freely available on the Web sites of PLoS Medicine at http://www.plosmedicine.org/, Annals of Internal Medicine at http://www.annals.org/, and Epidemiology at http://www.epidem.com/). Information on the STROBE Initiative is available at www.strobe-statement.org.

For peer review only
